# 20-HETE mediated TRPV1 activation drives allokinesis via MrgprA3^+^ neurons in chronic dermatitis

**DOI:** 10.7150/thno.85214

**Published:** 2024-02-04

**Authors:** Guang Yu, Pei Liu, Xiaobao Huang, Mingxin Qi, Xue Li, Weimeng Feng, Erxin Shang, Yuan Zhou, Changming Wang, Yan Yang, Chan Zhu, Fang Wang, Zongxiang Tang, Jinao Duan

**Affiliations:** 1 Jiangsu Collaborative Innovation Center of Chinese Medicinal Resources Industrialization, Jiangsu Key Laboratory for High Technology Research of TCM Formulae, Nanjing University of Chinese Medicine, Nanjing, China.; 2 Key Laboratory for Chinese Medicine of Prevention and Treatment in Neurological Diseases, School of Medicine, Nanjing University of Chinese Medicine, Nanjing, China.; 3 Department of Dermatology, The First Affiliated Hospital of Sun Yat-sen University, Guangzhou, China.

**Keywords:** 20-HETE, TRPV1, allokinesis, MrgprA3^+^ neurons, chronic dermatitis

## Abstract

**Rationale:** Noxious stimuli are often perceived as itchy in patients with chronic dermatitis (CD); however, itch and pain mechanisms of CD are not known.

**Methods:** TRPV1 involvement in CD was analyzed using a SADBE induced CD-like mouse model, and several loss- and gain-of-function mouse models. Trigeminal TRPV1 channel and MrgprA3^+^ neuron functions were analyzed by calcium imaging and whole-cell patch-clamp recordings. Lesional CD-like skin from mice were analyzed by unbiased metabolomic analysis. 20-HETE availability in human and mouse skin were determined by LC/MS and ELISA. And finally, HET0016, a selective 20-HETE synthase inhibitor, was used to evaluate if blocking skin TRPV1 activation alleviates CD-associated chronic itch or pain.

**Results:** While normally a pain inducing chemical, capsaicin induced both itch and pain in mice with CD condition. DREADD silencing of MrgprA3^+^ primary sensory neurons in these mice selectively decreased capsaicin induced scratching, but not pain-related wiping behavior. In the mice with CD condition, MrgprA3^+^ neurons showed elevated ERK phosphorylation. Further experiments showed that MrgprA3^+^ neurons from *MrgprA3;Braf* mice, which have constitutively active BRAF in MrgprA3^+^ neurons, were significantly more excitable and responded more strongly to capsaicin. Importantly, capsaicin induced both itch and pain in *MrgprA3;Braf* mice in an MrgprA3^+^ neuron dependent manner. Finally, the arachidonic acid metabolite 20-HETE, which can activate TRPV1, was significantly elevated in the lesional skin of mice and patients with CD. Treatment with the selective 20-HETE synthase inhibitor HET0016 alleviated itch in mice with CD condition.

**Conclusion:** Our results demonstrate that 20-HETE activates TRPV1 channels on sensitized MrgprA3^+^ neurons, and induces allokinesis in lesional CD skin. Blockade of 20-HETE synthesis or silencing of TRPV1-MrgprA3^+^ neuron signaling offers promising therapeutic strategies for alleviating CD-associated chronic itch.

## Introduction

Pain and itch are unpleasant sensations with cognitive, discriminatory, affective, and motivational components. These distinct somatosensory modalities elicit distinct behavioral responses, and are antagonistic - i.e. pain can inhibit itch 1, 2. Recent progress suggests that itch and pain are encoded and transmitted by discrete groups of primary sensory neurons (pruriceptive neurons and nociceptive neurons, respectively) in the trigeminal ganglia (TG) and dorsal root ganglia (DRG) 1, 2. However, the boundary between pain and itch becomes blurred in pathological conditions. For instance, iontophoresis of itch-inducing histamine is perceived as painful in patients with chronic pain 3, 4, while noxious mechanical, thermal and chemical stimuli are perceived as itchy in patients with chronic itch 5-7. The central or peripheral mechanisms of sensory switching is not known. It has been assumed that chronic itch leads to central sensitization of spinal itch circuits, so that inputs from peripheral nociceptive neurons are perceived as itchy rather than painful 6, 7. However, this theory is complicated by the discovery that most noxious stimuli, including capsaicin, activates pruriceptive neurons in addition to nociceptive neurons - suggesting that pain detection in the periphery stimuli also involve itch components 8-10.

Previous studies have identified several itch receptors among the Mas-related G protein-coupled receptors (Mrgprs) family, which includes more than 50 members in mice 8, 11, 12. Most Mrgprs are expressed solely by primary sensory neurons in the TG and DRG. MrgprA3 was identified as a receptor for chloroquine (CQ), an anti-malarial drug with serious pruritic side effects, and mediates histamine-independent itch induced by CQ 8. Interestingly, MrgprA3 is expressed by a highly restricted population of primary sensory neurons that accounts for about 5% of total DRG neurons 8. In addition to MrgprA3, this population also expresses high levels of histamine H1 receptors, serotonin receptors, proteinase activated receptors, as well as other Mrgprs, and can be activated by a variety of pruritogens. MrgprA3^+^ neurons selectively innervate skin in mice, and genetic ablation of this population results in severe defects in both acute and chronic itch. Conversely, specific activation of this population elicits robust itch-related scratching behavior. Together, these results indicate that MrgprA3^+^ neurons are itch selective primary sensory neurons 8, 11. Indeed, a recent study demonstrated that selective activation of these pruriceptive neurons using capsaicin, in the absence of nociceptive neurons activation, elicits itch 13.

Based on these previous reports, we hypothesized that selective sensitization of pruriceptive neurons occurs during chronic dermatitis (CD) condition, and hypersensitivity of itch sensing neurons suppress pain pathways. It is possible that the antagonistic relationship between pain and itch is bidirectional, depending on the relative activity of pain and itch pathways. However, it is unclear if or how MrgprA3^+^ neurons mediate itch and pain in chronic itch as hypothesized above. In this study, we examined the role of MrgprA3^+^ neurons under chronic itch conditions. Our results advance the understanding of abnormal sensory coding and dysregulated pain and itch crosstalk that occurs in CD condition, as well as provides critical information and therapeutic targets for itch management.

## Materials and methods

### Skin donors

Punch biopsies were obtained from healthy human skin and lesional skin of patients with CD, all of whom had active disease and did not receive any topical or systemic therapy for two or four weeks, respectively, before biopsy. Informed consent was obtained from all donors prior to biopsy and all procedures were approved by the Ethics Committee of the First Affiliated Hospital of Sun Yat-sen University (IRB number 2023 [068]).

### Animals

C57BL/6J mice, *Mrgpra3^Cre-Gfp+^*(*MrgprA3- Cre,* heterozygous for MrgprA3) mice, *Mrgpr-clusterΔ^-/-^* (*Mrgprs^-/-^*) mice, *B-raf^V600E^* mice (*Braf* mice), *Rosa26^tdTomato^* (*tdTomato*) mice and their WT littermate mice were used. Animals were bred as appropriate to generate: 1. *MrgprA3;Braf* mice, in which the constitutively active B-raf^V600E^ allele is specifically expressed in MrgprA3^+^ neurons; 2. *MrgprA3;tdTomato* reporter mice, in which the tdTomato fluorescent reporter is specifically expressed in MrgprA3^+^ neurons and nerve fibers; and 3. *MrgprA3;Braf;tdTomato* mice, in which both the constitutively active B-raf^V600E^ allele and the tdTomato fluorescent reporter are specifically expressed in MrgprA3^+^ neurons and nerve fibers.

Animal experiments were conducted in accordance with the relevant guidelines and regulations of the Institutional Animal Care and Use Committee of Nanjing University of Chinese Medicine (ACU190601). All mice used in this study were housed and tested in controlled environments with 20-24℃, 45-65% humidity, and 12-hour day/night light cycle. 8 to 10 week old male mice were used for behavioral tests, and littermate controls were used if possible. For our current study, we also generated the nape CD model using female mice to compare the sex-related differences on behavior. Researchers were blinded to animal genotypes and experimental groups throughout all manipulations, treatments, and data quantification.

### Generation of mouse CD model

CD model was induced using squaric acid dibutylester (SADBE) as reported previously 14. Briefly, mice were sensitized by topical application of 25 μL 1% SADBE in acetone onto their shaved abdominal skin once daily, starting on Day 1, for three consecutive days. Mice in the control group were treated with an acetone vehicle. Starting on Day 9, mice were challenged with topical application of 25 μL 0.5% SADBE in acetone onto their shaved nape or cheek skin once daily for five or three consecutive days. Control mice were challenged with acetone vehicle. Twenty-four hours after the challenge, scratching and wiping directed at the treated area were filmed and counted using criteria defined by our previous studies and relevant publications 15.

### Behavioral assay

Animals were acclimated to the testing environment for 15 minutes before the start of behavior tests. Scratching behavior in CD model animals were analyzed for 30 min immediately after habituation. Scratching behavior induced by subcutaneously injected CQ or capsaicin were observed for 30 min immediately after injection. A bout of scratching was defined as a continuous scratching movement with a hindlimb directed at the treatment or injection site, only those unilateral wipes with the forelimb directed at the treatment or injection site were defined as a continuous wiping movement 16. Drugs were administered as indicated in the figures, usually 24 hours after the third SADBE challenge. Drug dosages were selected based on pilot experiments or our previous studies or relevant studies 15, and are as follows: capsaicin (cheek, 500 µM, 20 µL; Sigma), chloroquine (cheek, 2 mM, 20 µL; Sigma), HET0016 (intraperitoneal, 2 mg/kg; Glpbio).

### DREADD studies

To genetically silence MrgprA3 neurons using the inhibitory, Gi-coupled hM4Di DREADD (designer receptors exclusively activated by designer drugs) receptor, hM4Di-mCherry adeno-associated virus (AAV) (Genomeditech, China) was injected by tail vein (10^11^ VG in 100 μL) into hemizygous *MrgprA3-Cre* and *MrgprA3;Braf* mice three weeks before the start of behavior experiments. 30 minutes after i.p. clozapine-N-oxide (CNO) injection (5 mg/mL, 100 μL/mouse), MrgprA3^+^ neurons are silenced and further capsaicin treatments only activate TRPV1^+^/MrgprA3^-^ neurons.

### TG neuron culture

TGs were dissected and collected in cold DH10 medium (90% DMEM/F-12, 10% FBS, 100 U/ml penicillin, 100 µg/ml streptomycin, Gibco) and digested with enzyme based dissociation solution (1 mg/mL Collagenase Type I and 5mg/mL Dispase in HBSS without Ca^2+^ and Mg^2+^, Gibco) at 37°C for 25-30 min with intermittent agitation. TG neurons were then freed by trituration through a pipette and large debris were removed using a cell strainer. Afterwards, neurons were pelleted by centrifugation, media was removed, and cells were re-suspended in warm (37°C) DH10 medium supplemented with nerve growth factor (NGF, 20 ng/ml) and glial cell line-derived neuro-trophic factor (GDNF) (25 ng/mL). Neurons were then seeded onto poly-D-lysine (0.5 mg/mL) and laminin (10 µg/mL) coated glass coverslips, cultured in an incubator (95% O_2_ and 5% CO_2_) at 37°C, and used within 24 hours.

### Ca^2+^ imaging

Calcium imaging was performed as previously described 17. In brief, cultured TG neurons were loaded with Fura-2 acetoxymethyl ester (Molecular Probes) for 30 min at room temperature. After washing and recovery for 5 min, cells were imaged at 340 and 380 nm excitation to detect free intracellular calcium. Neurons were considered responsive if ≥50% increase in baseline 340/380 fluorescence ratio was observed after stimulus administration. In Figure S3, TG neurons were loaded with Fluo-4 acetoxymethyl ester, neurons were imaged at 488-nm excitation to detect intracellular Ca^2+^ transients. In Figure S10, GFP signals from PirtGCaMP3-cultured neurons were imaged at 488-nm excitation to detect intracellular Ca^2+^ transients.

### Whole-cell patch-clamp recordings

MrgprA3^+^ neurons from *MrgprA3;tdTomato* and *MrgprA3;Braf; tdTomato* mice were first identified by red tdTomato fluorescence using wide field microscopy (ZEISS, Axio Oberver D1, Germany) and marked manually. Afterwards, coverslips were transferred into the electrophysiology recording chamber with extracellular solution. Whole-cell current clamp and voltage-clamp recording experiments were performed at room temperature (23 - 25°C) using a multi-clamp 700B amplifier and digital 1440 with pClamp10 software (Molecular Devices, USA) as previously described 17. Signals were sampled at 20 kHz and filtered at 2 kHz. Patch pipettes were pulled from borosilicate glass capillaries using a P-97 micropipette puller (Sutter Instrument) and all had resistances between 3 - 4 MΩ. Series resistance was routinely compensated at 60 - 80%. Resting membrane potential (RMP) was recorded for each neuron under the current-clamp mode after stabilization (within 3 min). Neurons with seal resistance below 1 GΩ were excluded from analysis. The liquid junction potential is 8 mV and corrected. To test AP firing, each neuron was injected with a series of depolarizing currents, 200ms duration and in 20 pA increments. Current threshold was defined as the minimum current required to induce an AP. Other AP-related parameters were measured in Clampfit software. Internal solution (in mM): KCl 135, MgATP 3, Na_2_ATP 0.5, CaCl_2_ 1.1, EGTA 2, Glucose 5, adjusted to pH 7.38 using KOH and 300 mOsm with sucrose. External solution (in mM): NaCl 140, KCl 4, CaCl_2_ 2, MgCl_2_ 2, HEPES 10, Glucose 5, adjusted to pH 7.4 using NaOH and 310 mOsm with sucrose.

### Western blot

Western blotting was performed as described previously 17. Briefly, fresh tissues were homogenized in RIPA buffer (P0013B, Beyotime, China) in accordance with the manufacture's protocols. Protein concentration was determined by BCA assay. 60 μg of protein was loaded per lane and resolved on 12% SDS-polyacrylamide gels. Polyclonal anti-pERK antibody (#4370, Cell signaling technology) and monoclonal GAPDH antibody (ab8245, Abcam) were both used at 1:1000 dilution. Primary antibodies were detected using HRP-conjugated goat anti-rabbit antibodies and a chemiluminescence kit (Tanon, Shanghai, China). Band intensities were quantified using ImageJ software.

### Untargeted metabolomics analyses

Total protein lysate from skin were prepared using RIPA Lysis Buffer and protein concentration was determined by BCA assay. Samples were desalted using Ziptip C18 columns before LC-MS analysis. UPLC-Q-TOF/MS analysis was performed using an UHPLC Acquity™ system (Waters Corp., Milford, MA, USA) coupled to a Synapt™ Q-TOF mass spectrometer with electrospray ionization (ESI) in positive and negative modes. Phase separation was performed on an ACQUITY UPLC BEH-C18 chromatographic column (2.1 × 100 mm, 1.7 μm) at 30°C. 2 μL of each sample was injected and flow rate of the mobile phase was set to 0.4 mL/min. Acetonitrile (ACN) gradient was increased stepwise from 0% with 0.1% formic acid to 95% over 20 minutes as follows: 0-2.5 min, 5-35 % ACN; 2.5-4 min, 35-45 % ACN; 4-10 min, 45-65 % ACN; 10-11 min, 65-85 % ACN; 11-16 min, 85-90% ACN; 16-18 min, 90-95% ACN; 18-19 min, 95% ACN; 19-19.5 min, 95-5% ACN; 19.5-20 min, 5% ACN.

To maximize metabolite identification, mass data were acquired for m/z 100 to 1000 in both the negative and positive ESI modes. Data were acquired using the MSE method using low (6 eV) and high (20-60 eV) energy functions for precursor and fragments, respectively. Calibration was performed using leucine-enkephalin (ESI^+^: m/z 556.2771, ESI^-^: m/z 554.2615). Optimizations include: 140°C ion source temperature, 800 L/h 400°C nitrogen desolvation gas, 50 L/h cone gas, 0.15 mL/min collision gas, 3 kV capillary voltage in the positive mode and 2.5 kV in the negative mode, 30 V cone voltage, 1 V ion guide, and 40 psi nebulizer pressure.

Raw data were processed using MassLynx™ v4.1 workstation software (Waters Corp.) for metabolic fingerprint profiling including peak detection, noise removal, filtering, and alignment. Processed data matrix includes retention time (tR), m/z value, and normalized ion intensity for each peak area.

Endogenous metabolites with variable projection importance (VIP) value in OPLS-DA greater than 1 and t-test *P* value < 0.05 were considered differential metabolites associated with CD. The differential metabolites were identified by combining and comparing with Human Metabolome Database (HMDB: https://hmdb.ca/) and other databases. These metabolites were introduced into Metaboanalyst 5.0 for analyzing their related metabolic pathways (https://www.metaboanalyst.ca/) 18. Impact values present the topology structure's importance of metabolites in their corresponding metabolic pathways. The higher the impact value, the greater the structure's importance of metabolites involved in this pathway 19.

### 20-HETE assay

The UHPLC analysis was performed using a Dionex Ultimate 3000 UHPLC system (Thermo Scientific, USA). Separation was performed using an Acquity UPLC CSH-C18 column (2.1 mm × 100 mm, 1.7 μm, Waters, Milford, USA). 2 μL of each sample was injected and flow rate of the mobile phase was set to 0.4 mL/min. Linear gradient elution was performed with 0.1% formic acid / water (v/v, solvent A) and acetonitrile (solvent B), and separation was carried out over 30 min with gradually increasing ACN: 0-7min, 5%-10% ACN; 7-10 min, 10%-20% ACN; 10-12 min, 20% ACN; 12-14 min, 20%-45% ACN; 14-21min, 45%-80% ACN; 21-27 min, 80%-100% ACN; 27-28.5 min, 100%-5% ACN; 28.5-30 min, 5%. Column temperature was maintained at 30°C. Mass spectra was acquired for m/z 100 to 1000 Da in centroid mode using an LTQ-Orbitrap Velos mass spectrometer (Thermo Scientific, USA) with ESI, and processed using XcaliburTM v2.2 (Thermo Scientific, USA). Optimizations include: 3.8 kV spray voltage, > 99.99% pure nitrogen sheath gas and auxiliary gas, > 99.99% pure helium collision gas, sheath gas flow rate 25 AU, auxiliary gas flow rate 15 AU, 350°C ion source temperature, 320°C capillary temperature.

20-HETE concentration in skin lysates of HET0016 treated mice were measured by 20-HETE enzyme-linked immunosorbent assay (ELISA) kit (mlbio, Shanghai, China) according to the manufacturer's instruction.

### Immunofluorescence

Immunofluorescent imaging of TG and skin tissue were performed as previously described 20. Briefly, mice were anesthetized with 1% sodium pentobarbital (50 mg/kg, i.p.) and transcardially perfused with cold 0.1 M phosphate-buffered saline (PBS, pH 7.4, 4℃) followed by 4% paraformaldehyde in PBS (pH 7.4, 4°C). TG and skin were then dissected and cryoprotected in 30% sucrose at 4°C for 24 hours. Afterwards, dissected tissues were embedded in optimum cutting temperature compound (OCT, Leica, Wetalar, Germany) and rapidly frozen at -20°C (CM1950, Leica). TGs and skin were sectioned at 10 μm or 50 μm thickness, respectively, and slide mounted using a sliding microtome (CM1950, Leica). Slides were then incubated in blocking solution (10% fetal bovine serum in PBS containing 0.1% Triton X-100) for 30 min at room temperature, followed by primary antibody at 4°C overnight and in secondary antibody at room temperature for 2 hours in the dark. Images were acquired using an Olympus fluorescence microscope (BX51, Olympus Japan).

### Data analysis

All experiments were performed by researchers blinded to animal genotype or experimental group. Experimental groups, treatment order, and testing order were assigned using simple randomization. Data are presented as the mean ± SEM. Data analysis was performed using GraphPad Prism 8.0 software (GraphPad Software Inc., San Diego, CA, USA). For statistical comparisons between 2 groups, either unpaired Student's t test (2-tailed) or Mann-Whitney U test with Welch's correction was used, depending on normality of data distribution. One-way analysis of variance (ANOVA) was used for the comparisons among multiple groups. Difference was considered statistically significant if *P* < 0.05.

## Results

### TRPV1 mediated itch is selectively enhanced in the SADBE-induced CD model mouse

Squaric acid dibutylester (SADBE), a small molecule hapten, has commonly been used to induce non-allergic and allergic CD in mice and, in a similar fashion, an area of contact hypersensitivity (CHS). Similar to previous reports 14, our SADBE nape model induced significant spontaneous scratching behavior in mice (Figure S1A-B). To examine the effect of this model on pain and itch sensation, we further tested the acute effects of CQ or capsaicin 24 hours after two challenges (Figure S1C). Both CQ and capsaicin induced significant scratching response in mice (Figure S1D). Moreover, compare to vehicle control treated mice, scratching responses to both CQ (50 ± 11 SADBE vs. 24 ± 3.6 Control) and capsaicin (64 ± 10.5 SADBE vs. 28 ± 6.6 control) were enhanced in CD mice (Figure S1E). The enhanced scratching behavior induced by capsaicin was also observed in CD female mice (Figure S1F).

**Figure 1:**
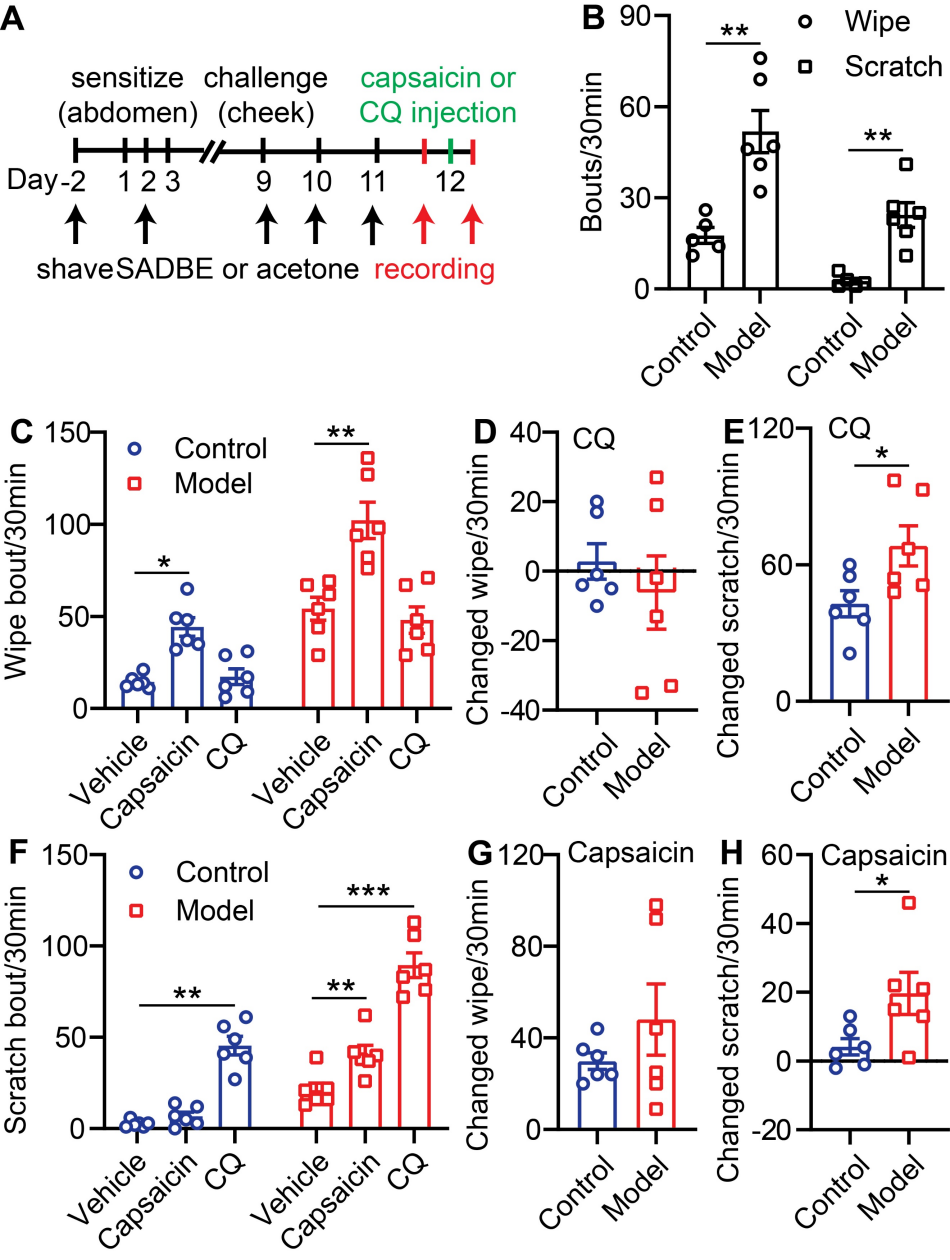
** TRPV1 mediated itch is selectively enhanced in the SADBE-induced CD mouse model. (A)** Schematic of the SADBE induced CD model and time points of drug injections. **(B)** Spontaneous wiping and scratching behavior in vehicle treated control and SADBE treated CD model mice. (n = 6). **(C)** Capsaicin (500 µM/20 µL) or CQ (2 mM/20 µL) induced wiping in control and CD model mice. (n = 6). **(D)** The change in wiping response after CQ injection in control mice (the number of CQ-induced wiping in control group - the number of vehicle-induced wiping in control group) and CD model mice (the number of CQ-induced wiping in SADBE model group - the number of vehicle-induced wiping in SADBE model group). **(E)** The change in scratching after CQ injection in control mice (the number of CQ-induced scratching in control group - the number of vehicle-induced scratching in control group) and CD model mice (the number of CQ-induced scratching in SADBE model group - the number of vehicle-induced scratching in SADBE model group). **(F)** Capsaicin (500 µM/20 µL) or CQ (2 mM/20 µL) induced scratching behavior in control and CD model mice. (n = 6). **(G)** The change in wiping response after capsaicin injection in control mice (the number of capsaicin-induced wiping in control group - the number of vehicle-induced wiping in control group) and CD model mice (the number of capsaicin-induced wiping in SADBE model group - the number of vehicle-induced wiping in SADBE model group). **(H)** The change in scratching after capsaicin injection in control mice (the number of capsaicin-induced scratching in control group - the number of vehicle-induced scratching in control group) and CD model mice (the number of capsaicin-induced scratching in SADBE model group - the number of vehicle-induced scratching in SADBE model group). (n = 6). *, *P* < 0.05; **, *P* < 0.01; ***, *P* < 0.001. All data are presented as mean ± SEM.

To further validate the pain and itch effects of CQ or capsaicin in CD, we generated a cheek model where pain and itch behavioral responses can be differentiated by forelimb wiping (pain) or hindlimb scratching (itch) (Figure S2A) 16. In this model, SADBE treatment induced both scratching (21 ± 3.8 SABDE vs. 2.6 ± 0.9 Control) and wiping behavior (54 ± 6.3 SADBE vs. 18 ± 2.7 Control) in mice after 3 challenges (Figure 1A-B). CQ injection specifically increased scratching, but not wiping, behavior in both control and CD model mice (Figure 1C and F). Moreover, compared to control mice, CQ induced scratching was especially enhanced in CD model mice (68 ± 8.9 SADBE vs. 43 ± 5.7 Control) (Figure 1E), while no difference in wiping responses was detected (Figure 1D). This finding is consistent with common reports of hyperkinesis in chronic itch patients 21. Vehicle injection did not produce any detectible effects on scratching or wiping behavior in CD model mice (Figure S2B). Interestingly, change in capsaicin induced wiping behavior was not significantly different between control and CD model mice (48 ± 16.5 SADBE vs. 30 ± 4.2 Control) (Figure 1C, G), but capsaicin only induced scratching in CD mice (20 ± 1.8 SADBE vs. 4 ± 2.4 Control) (Figure 1F, H). This observation is consistent with previous reports, that chronic itch enhanced excitability of nociceptors 15, 22.

### MrgprA3^+^ neurons are required for TRPV1 mediated itch in CD condition

**Figure 2:**
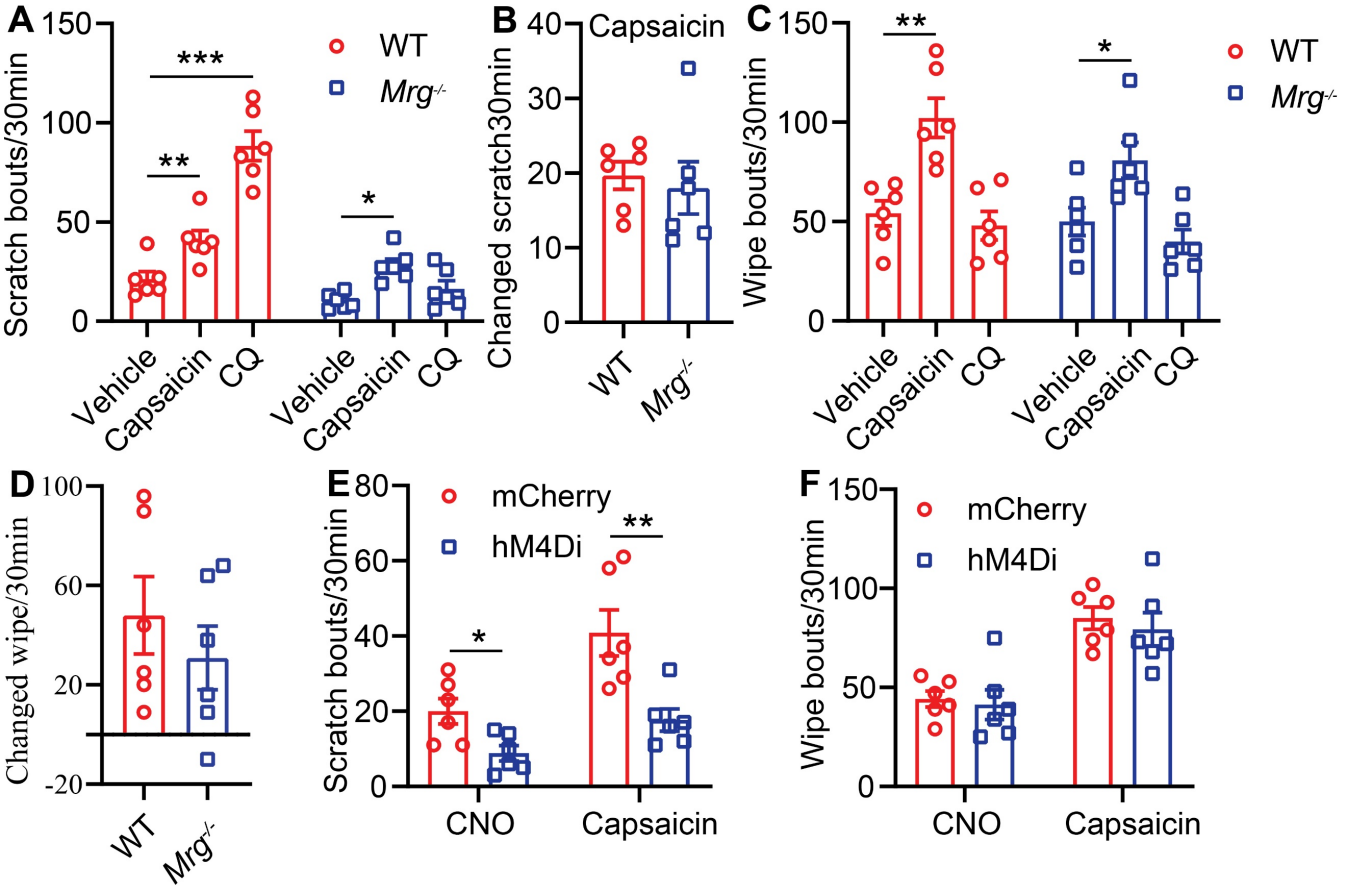
** MrgprA3^+^ neurons are required for TRPV1 mediated itch in CD condition. (A)** Capsaicin (500 µM/20 µL) or CQ (2 mM/20 µL) induced scratching behavior in WT and *Mrgprs^-/-^* mice with CD condition. (n = 6). **(B)** The change in scratching after capsaicin injection in WT and *Mrgprs^-/-^* mice with CD condition. **(C)** Capsaicin (500 µM/20 µL) or CQ (2 mM/20 µL) induced wiping behavior in WT and *Mrgprs^-/-^* mice with CD condition. (n = 6). **(D)** The change in wiping after capsaicin injection in WT and *Mrgprs^-/-^* mice with CD condition. **(E-F)** Spontaneous and capsaicin induced scratching **(E)** and wiping **(F)** behavior in CNO treated (30 min pre) control and MrgprA3-hM4Di mice with CD condition. The changed behavior analysis method is consistent with Figure 1. *, *P* < 0.05; **, *P* < 0.01; ***, *P* < 0.001. All data are presented as mean ± SEM.

Although TRPV1 was reported to contribute to MrgprA3^+^ mediated itch sensations 13, the involvement Mrgprs (including MrgprA3) in capsaicin induced itch and pain in CD disorder has not been tested. To examine this possibility, we generated the CD model using *Mrgprs^-/-^* mice. Mrgprs deficiency resulted in significantly decreased scratching behavior in the CD model (10 ± 1.6 *Mrgprs^-/-^* mice vs. 21 ± 3.8 WT) (Figure 2A) without affecting pain related wiping behavior (Figure 2C). Moreover, CQ injection enhanced scratching in WT, but not *Mrgprs^-/-^* CD model mice (Figure 2A), without effects in wiping behavior (Figure 2C). Remarkably, capsaicin enhancement of wiping and scratching were similar in WT and *Mrgprs^-/-^* CD model mice (Figure 2A-D), suggesting that Mrgprs are not required for capsaicin induced pain or itch in the CD model mice.

To further test if MrgprA3^+^ neurons are required for itch and pain in the mouse CD model, we examined the effects of DREADD mediated genetic silencing of MrgprA3^+^ neurons. To accomplish this, we specifically introduced the inhibitory Gi-coupled hM4Di receptor into MrgprA3^+^ neurons using AAV and *MrgprA3-Cre* mice. CQ induced calcium response in cultured TG neurons was significantly decreased in the hM4Di group after CNO pretreatment (Figure S3 A-B). CQ induced scratching behavior was significantly decreased in the hM4Di group after i.p. CNO pretreatment (Figure S3 C), without detectible differences in either CQ or capsaicin induced wiping (Figure S3 D). Moreover, CNO specifically inhibited CD-associated spontaneous scratching in the hM4Di group (Figure 2E, left panel) without effects on spontaneous CD-associated wiping (Figure 2F, left panel). Importantly, in the CD model mice, only capsaicin induced scratching (Figure 2E, right panel), but not wiping (Figure 2F, right panel), was significantly inhibited by CNO pretreatment. Together, our results demonstrate that while Mrgprs are required for CD-associated chronic itch, MrgprA3^+^ neurons, but not Mrgpr receptors, are required for capsaicin induced allokinesis in SADBE model.

### ERK phosphorylation is enhanced in MrgprA3^+^ neurons in CD condition

**Figure 3:**
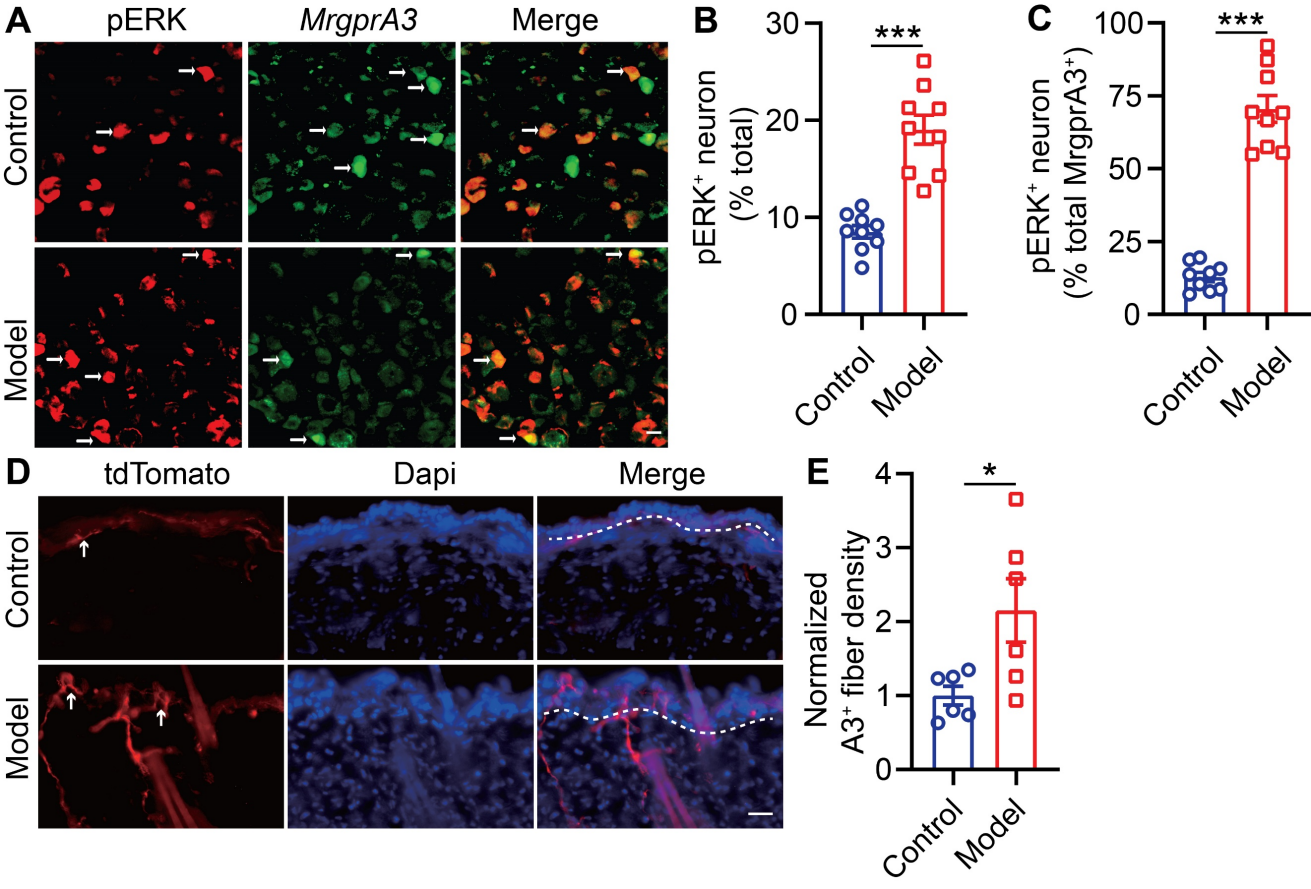
** ERK phosphorylation and MrgprA3^+^ skin fiber density are enhanced in CD condition. (A)** Immunostaining of pERK in TGs from *MrgprA3-Cre* mice with CD condition (left panel: arrows indicate pERK^+^ neurons; middle panel: arrows indicate MrgprA3^+^ neurons; right panel: arrows indicate double positive neurons). **(B)** Quantification of the proportion of pERK^+^ neurons in TGs from mice with CD condition. (n = 3). **(C)** Quantification of pERK positivity in MrgprA3^+^ TG neurons in mice with CD condition. **(D)** Immunostaining of MrgprA3-tdTomato in control and lesional skin of CD model mice. The thickness of slice is 50 μm. Above the dotted line is the epidermis. **(E)** Quantification of MrgprA3^+^ fiber density in control and lesional skin of CD model mice. The scale bar represents 25 µm. *, *P* < 0.05; ***, *P* < 0.001. All data are presented as mean ± SEM.

Raf/MEK/ERK (MAPK) signaling regulates the expression and activity of a plethora of transcription factors and exerts actions across a wide array of cellular functions. One previous study had reported that U0126, a MEK inhibitor, attenuated scratching in a chronic itch model 23. To study if MAPK elevated ERK phosphorylation signaling contributes to MrgprA3^+^ neuron mediated in CD-like disease, we examined activated ERK (pERK) in MrgprA3^+^ TG neurons of CD model mice (Figure 3A). After the third SADBE challenge, the percentage of pERK^+^ neurons in the TG was increased from 8.5 ± 0.6 (control) to 19.0 ± 1.5 (CD model) (Figure 3B). Importantly, the percentage of pERK and MrgprA3 double-positive neurons was significantly increased in CD model mice (12.9 ± 1.6 control vs. 70.5 ± 4.6 CD model mice) (Figure 3C). In addition to pERK, nerve fiber density in the skin is often increased in chronic itch skin condition, which may also contribute to enhanced itch and itch detection. To test if this is another parallel mechanism of CD-associated itch, the length of MrgprA3^+^ sensory fibers per square millimeter was measured in skin of control and CD model mice. Indeed, the density of MrgprA3^+^ fiber in skin was significantly increased (Figure 3D-E).

### ERK phosphorylation is enhanced in *MrgprA3;Braf* mice

**Figure 4:**
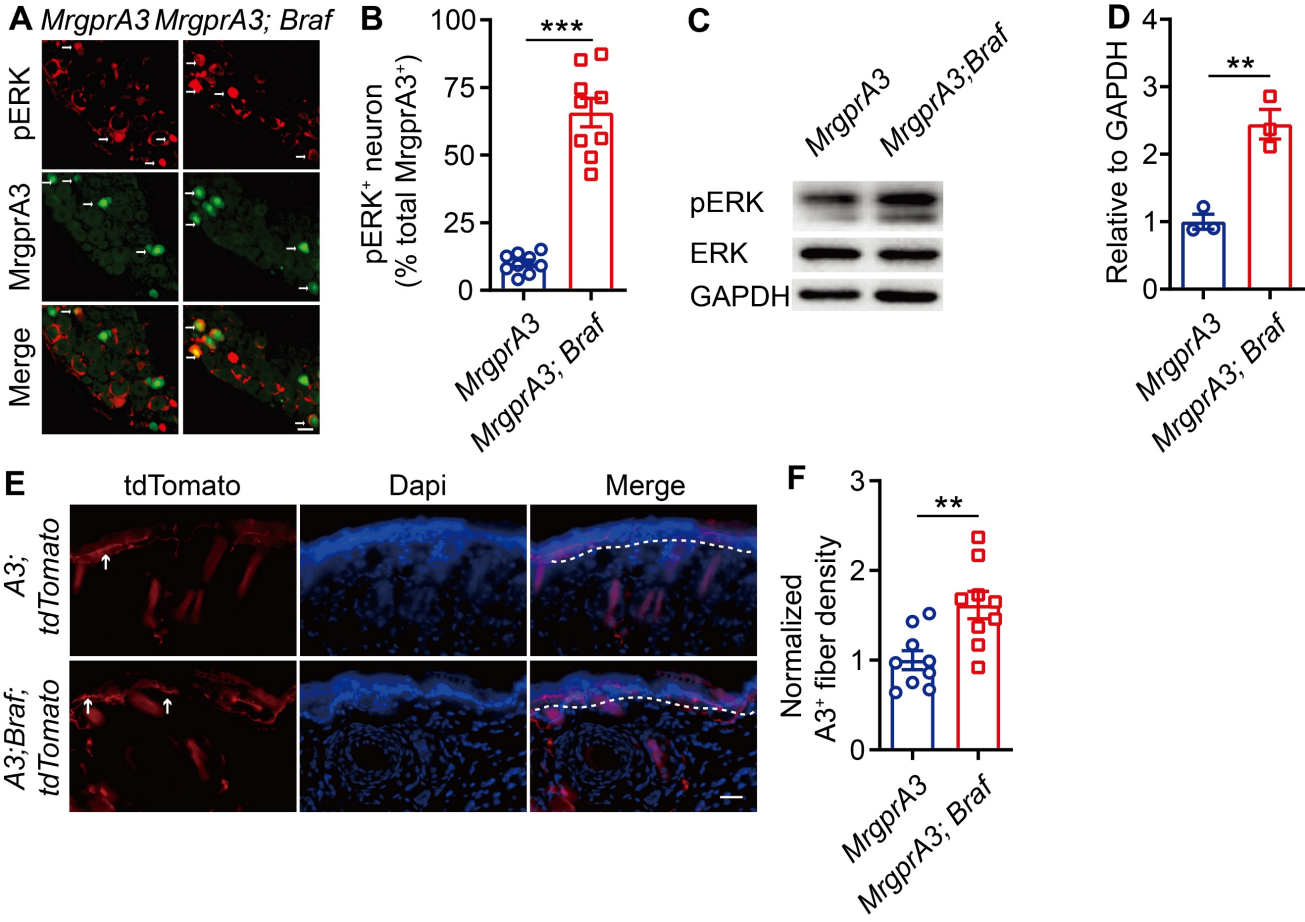
** ERK phosphorylation and MrgprA3^+^ skin fiber density are elevated in *MrgprA3;Braf* mice. (A)** Immunostaining of pERK in TGs from *MrgprA3-Cre* and *MrgprA3;Braf* mice (top panel: arrows indicate pERK^+^ neurons; middle panel: arrows indicate MrgprA3^+^ neurons; bottom panel: arrows indicate double positive neurons). **(B)** Quantification of pERK positivity in MrgprA3^+^ TG neurons from *MrgprA3-Cre* and *MrgprA3;Braf* mice. **(C)** Representative western blot bands showing pERK, total ERK, and GAPDH loading control in TGs from *MrgprA3-Cre* and *MrgprA3;Braf* mice.** (D)** Quantification of western blot bands from the previous panel, showing normalized pERK intensity in TGs of *MrgprA3-Cre* and *MrgprA3;Braf* mice. (n = 3). **(E)** Immunostaining of tdTomato in skin from *MrgprA3;tdTomato* and *MrgprA3;Braf;tdTomato* mice. The thickness of slice is 50 μm. Above the dotted line is the epidermis. **(F)** Quantification of MrgprA3^+^ fiber density in skin. The scale bars represent 50 µm. **, *P* < 0.01; ***, *P* < 0.001. All data are presented as mean ± SEM.

BRAF is a serine/threonine kinase that activates ERK, a member of the MAPKs superfamily, through the RAF/MEK/ERK cascade 24. To mimic the increased ERK phosphorylation and excitability of MrgprA3^+^ neurons in our CD model, and to investigate MrgprA3^+^ neuron functions in itch and pain, we crossed *MrgprA3-Cre* mice with *Braf* mice. In the resulting *MrgprA3;Braf* mice, constitutively active Braf is specifically expressed in MrgprA3^+^ neurons (Figure S4). Activated Braf activates MEK, which in turn activates ERK. Consistent with expectations, the percentage of pERK^+^ MrgprA3^+^ neurons was significantly increased in *MrgprA3;Braf* mice (10.0% in control *MrgprA3-Cre* mice vs 65.7% in* MrgprA3;Braf* mice, Figure 4A-B). Furthermore, ERK phosphorylation was significantly elevated in the TGs of *MrgprA3;Braf* mice, compared to *MrgprA3-Cre* mice (Figure 4C-D). Importantly, even though Braf activity was increased in MrgprA3^+^ neurons of* MrgprA3;Braf* mice, the proportion of other population markers, including CGRP and NF200, were not different between *MrgprA3-Cre* and *MrgprA3;Braf* mice - suggesting that fates of peptidergic (Figure S5A-B) and large-diameter (Figure S5C-D) neurons were preserved in *MrgprA3;Braf* mice. Because GFP fluorescence of the Cre-GFP protein is nearly completely localized to the nucleus in *MrgprA3-Cre* mice, we further generated *MrgprA3;tdTomato* and* MrgprA3;Braf; tdTomato* mice (Figure S4), so that MrgprA3^+^ fibers in the skin can be visualized using the red tdTomato fluorescent reporter. Imaging showed that the innervation density of MrgprA3^+^ sensory fibers was increased by 60% in *MrgprA3;Braf; tdTomato* mice, compared to *MrgprA3;tdTomato* mice (Figure 4E-F, Figure S6).

### MrgprA3^+^ neurons from *MrgprA3;Braf* mice show increased excitability

**Figure 5:**
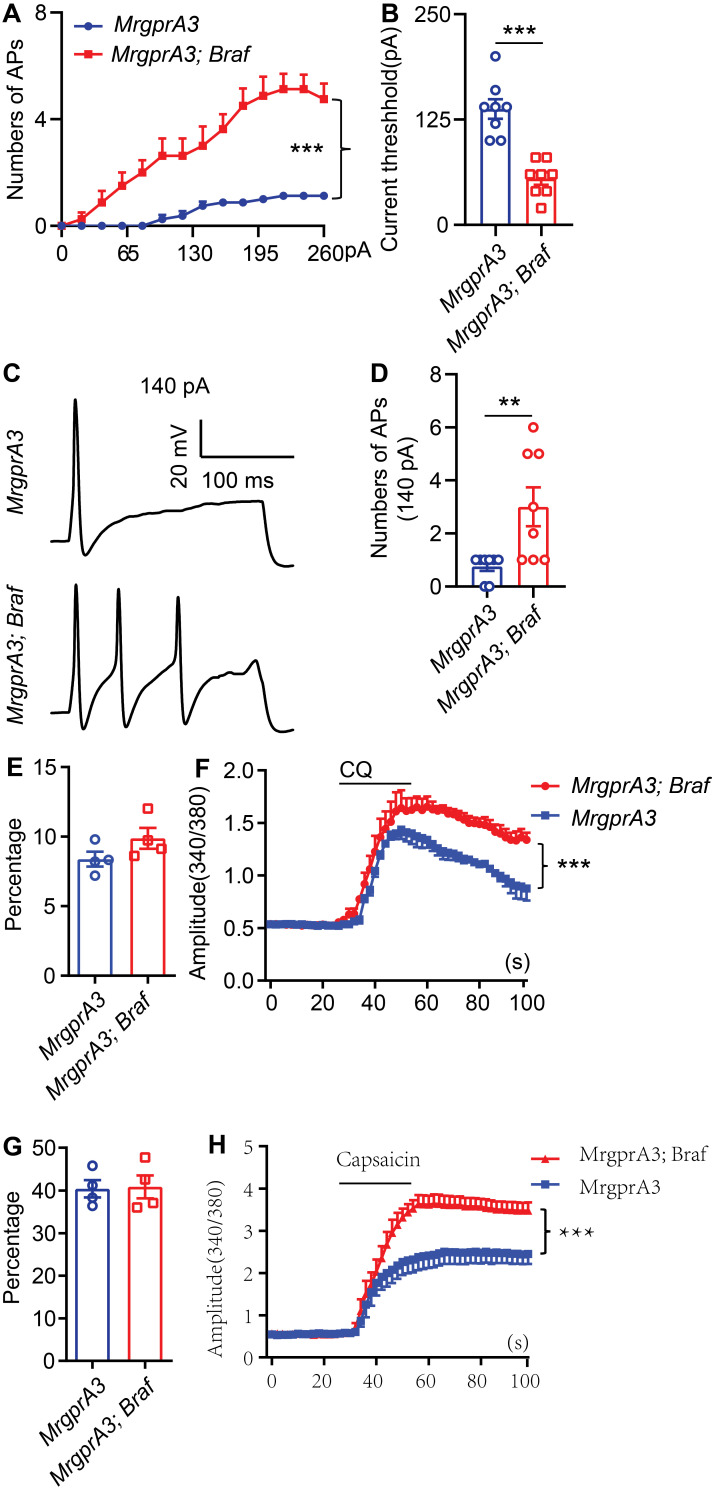
** MrgprA3^+^ neurons from *MrgprA3;Braf;tdTomato* mice show increased excitability. (A)** Current induced AP firings in MrgprA3^+^ TG neurons from *MrgprA3;tdTomato* and *MrgprA3;Braf;tdTomato* mice. **(B)** Threshold current required to induce the first AP in MrgprA3^+^ TG neurons from *MrgprA3;tdTomato* and *MrgprA3;Braf;tdTomato* mice. (n = 8). **(C)** Representative 140 pA induced APs in MrgprA3^+^ TG neurons from *MrgprA3;tdTomato* and* MrgprA3;Braf;tdTomato* mice. **(D)** Quantification of the number of 140pA induced APs in MrgprA3^+^ TG neurons from *MrgprA3;tdTomato* and *MrgprA3;Braf;tdTomato* mice. (n = 8). **(E)** The percentage of CQ responsive TG neurons in *MrgprA3-Cre* and *MrgprA3;Braf* mice. (n = 4).** (F)** CQ induced calcium responses (averaged trace) in TG neurons from *MrgprA3-Cre* and *MrgprA3;Braf* mice. The calcium responses difference between two kinds of mice were compared by the area under the curve. (n = 4). **(G)** The percentage of capsaicin responsive TG neurons in *MrgprA3-Cre* and *MrgprA3;Braf* mice. (n = 4). **(H)** Capsaicin induced calcium responses (averaged trace) in TG neurons from *MrgprA3-Cre* and *MrgprA3;Braf* mice. The calcium responses difference between two kinds of mice were compared by the area under the curve. (n = 4). **, *P* < 0.01; ***, *P* < 0.001. All data are presented as mean ± SEM.

To test if increased pERK signaling in MrgprA3^+^ neurons results in enhanced excitability of this population, we performed whole cell patch clamp recordings of cultured TG neurons from *MrgprA3;tdTomato* and* MrgprA3;Braf; tdTomato* mice. MrgprA3^+^ neurons were identified by tdTomato fluorescence and recorded in current clamp mode. Each neuron was injected with a train of 200 ms currents, increasing from 0 to 260 pA in 20 pA increments, to evoke action potentials (APs) firing. While MrgprA3^+^ neurons from *MrgprA3;tdTomato* mice only generated a few APs, most of which were single spikes, those from *MrgprA3;Braf; tdTomato* mice fired significantly more APs and frequently generated multiple spikes (Figure 5A). Moreover, the minimum current required to evoke AP was significantly lower in MrgprA3^+^ neurons from *MrgprA3;Braf; tdTomato* mice than those from control mice (137 ± 11.6 pA in *MrgprA3;tdTomato* mice vs 55 ± 7.3 pA in *MrgprA3;Braf; tdTomato* mice, Figure 5B). Specifically with 140 pA currents, MrgprA3^+^ neurons from *MrgprA3;tdTomato* mice produced single spike or no firing, while those from *MrgprA3;Braf; tdTomato* mice generated an average of 3 spikes (Figure 5C-D).

Calcium imaging experiments further confirmed that CQ and capsaicin responsiveness were enhanced in MrgprA3^+^ neurons of *MrgprA3;Braf* mice. While the percentage of CQ responsive neurons was not changed (Figure 5E), CQ induced calcium amplitudes were significantly increased in MrgprA3^+^ neurons from *MrgprA3;Braf* mice, compared with those from the *MrgprA3-Cre* mice (Figure 5F). Importantly, capsaicin induced calcium amplitudes were also significantly increased in MrgprA3^+^ neurons from* MrgprA3;Braf* mice (Figure 5G-H).

### TRPV1 mediated itch, but not pain, is selectively enhanced in *MrgprA3;Braf* mice

**Figure 6:**
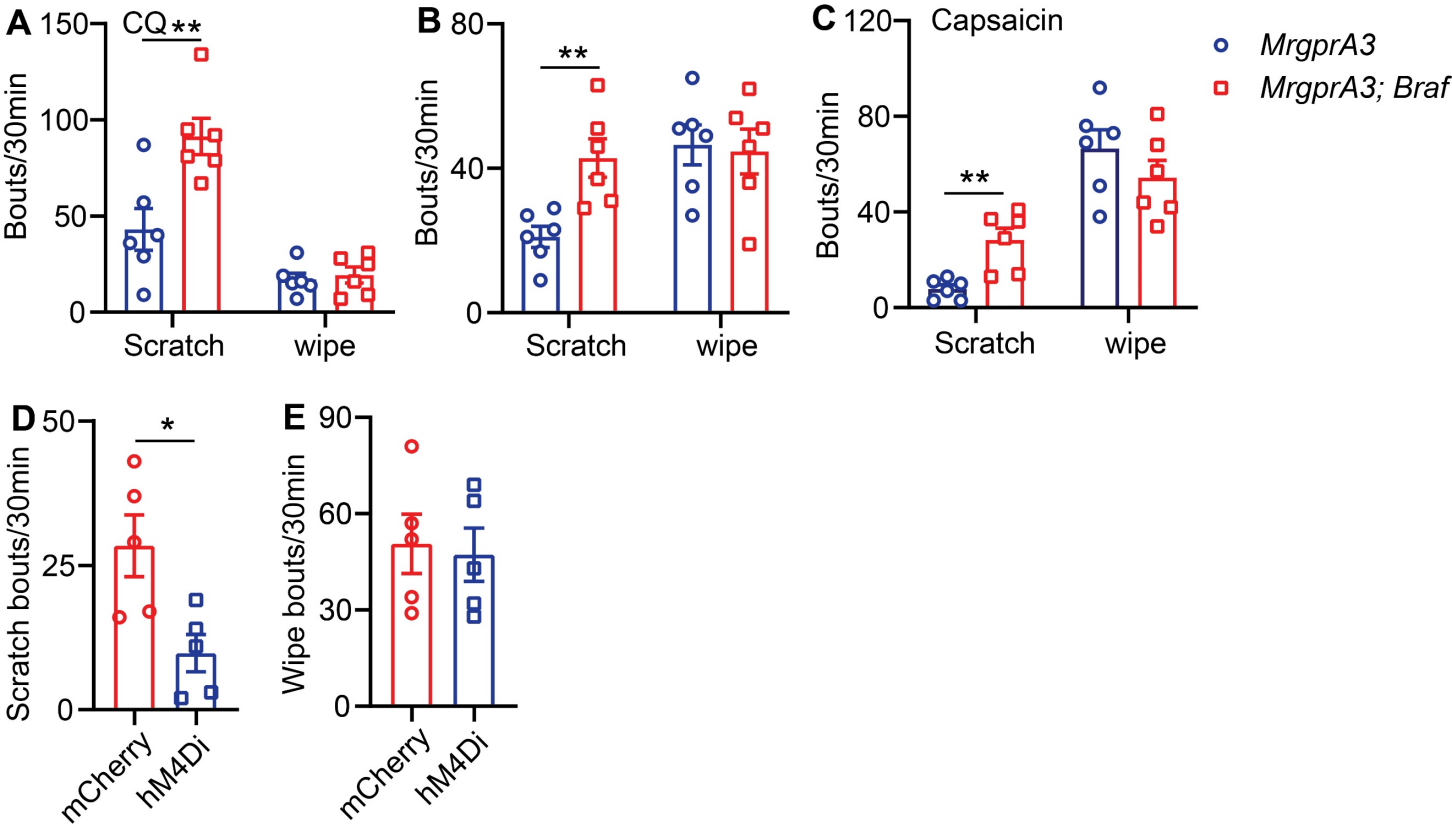
** TRPV1 mediated itch, but not pain, is selectively enhanced in *MrgprA3;Braf* mice. (A)** CQ (2 mM/20 µL) induced scratching behavior in *MrgprA3-Cre* and *MrgprA3;Braf* mice. (*, compared with *MrgprA3-Cre* mice; n = 6). **(B)** Spontaneous scratching and wiping behavior in control and *MrgprA3;Braf* mice with CD condition. (n = 6). **(C)** Capsaicin (500 µM/20 µL) induced scratching and wiping behavior in *MrgprA3-Cre* and *MrgprA3;Braf* mice. (n = 6). **(D)** Capsaicin (500 µM/20 µL) induced scratching behavior in control mCherry AAV injected and hM4Di AAV injected *MrgprA3;Braf* mice after CNO pretreatment. (n = 5). **(E)** Capsaicin (500 µM/20 µL) induced wiping behavior in control mCherry AAV injected and hM4Di AAV injected *MrgprA3;Braf* mice after CNO pretreatment. (n = 5). *, *P* < 0.05; **, *P* < 0.01. All data are presented as mean ± SEM.

Next, we assessed the consequences of enhanced pERK signaling and excitability of MrgprA3^+^ neurons on itch and pain. We first tested if CQ induced acute itch was altered in *MrgprA3;Braf* mice, and found that scratching, but not wiping, was significantly increased compared to control mice (Figure 6A). Selective itch enhancement was also found in the CD model. After SADBE treatments, *MrgprA3;Braf* mice scratched significantly more than control *MrgprA3-Cre* mice (43 ± 5.3 vs 21 ± 3.0, Figure 6B), while no difference in wiping was found (Figure 6B). Remarkably, selective itch enhancement was also observed in the *MrgprA3;Braf* mice after capsaicin injection. Capsaicin injection acutely and potently induces burning pain in naïve mice 15. While capsaicin induced acute pain was not different between *MrgprA3;Braf* and *MrgprA3-Cre* mice, *MrgprA3;Braf* mice scratched in response to the capsaicin injections (28 ± 5.0* MrgprA3;Braf* vs. 8 ± 1.7* MrgprA3-Cre* mice, Figure 6C) - indicating that enhanced MrgprA3^+^ neuron excitability leads to itch hypersensitivity. This itch response was attenuated by DREADD mediated silencing of MrgprA3^+^ neurons. Similar to our findings with the wild-type CD model mice (Figure 2E), capsaicin induced scratching in *MrgprA3;Braf* mice was significantly inhibited by CNO pre-treatment (Figure 6D), without detectible effects on pain responses (Figure 6E). Additional von Frey and Hargreaves assays also did not detect any changes in acute pain responses in the *MrgprA3;Braf* mice (Figure S7A-B).

### The arachidonic acid metabolite and TRPV1 agonist 20-HETE is enriched in lesional skin of CD model mice

**Figure 7:**
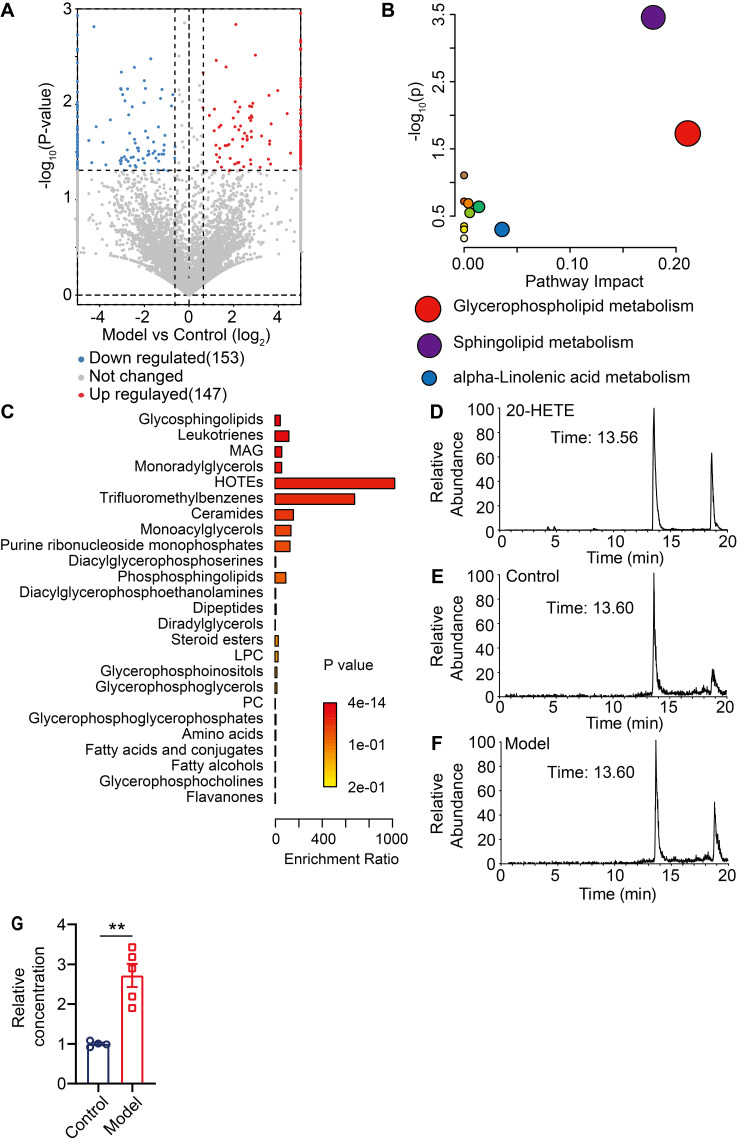
** Unbiased metabolomic analysis of lesional skin of CD model mice. (A)** Volcano plot highlighting differentially abundant metabolites in lesional CD model skin and control skin comparison, presented as -log_10_ (P-value) of CD/control. **(B)** Pathway analysis of differentially abundant compounds. **(C)** Enrichment analysis of differentially available compounds. **(D)** UHPLC ion chromatogram of the reference 20-HETE compound. **(E-F)** Ion chromatograms of 20-HETE in control and lesional CD skin lysates.** (G)** Normalized 20-HETE abundance in control and lesional CD skin. **, *P* < 0.01. All data are presented as mean ± SEM.

Thus far, our data has demonstrated that pERK signaling is elevated in MrgprA3^+^ neurons in CD condition, which leads to increased excitability of MrgprA3^+^ neurons and augmented TRPV1 mediated itch. Next, we set to identify endogenous TRPV1 ligands that are produced by lesional CD skin after the third SADBE challenge. To accomplish this, we analyzed skin lysates of CD model mice using UHPLC MS/MS and identified 147 compounds with increased abundance and 153 with decreased abundance in lesional skin, compared to vehicle treated skin (Figure S8 and Figure 7A). Analysis of the 175 compounds with VIP values greater than 1 further showed that the most significant changes were concentrated in phospholipid metabolism pathways (Figure 7B), and that most of the differentially abundant metabolites were associated with glycerophospholipid metabolism (Figure 7C). Among the identified compounds, arachidonic acid metabolites were especially promising candidates as these compounds are components of glycerophospholipids with broad bioavailability and had been previously implicated in inflammation as well as pain and itch processes. One metabolite, 20-hydroxyeicosatetraenoic acid (20-HETE), had previously been reported to activate TRPV1 25. Based on these reasons, we further quantified 20-HETE availability in the skin by UHPLC (Figure 7D-F). Reference 20-HETE compound (Cayman) was used to determine retention time (t_R_ = 13.56 min) and precursor ion (m/z = 338.34) in our LC-MS system (Figure 7D and Figure S9A). 20-HETE was found in both control and lesional CD skin (Figure 7E-F and Figure S9B-C). Due to significant thickening of the lesional skin samples, further normalization revealed that the relative concentration of 20-HETE was significantly increased in lesional skin of CD model mice (Figure 7G).

### 20-HETE mediated TRPV1 activation drives CD-associated itch

Due to the high abundance of 20-HETE in lesional skin of our CD model mice, as revealed by UHPLC, and its potency in activating TG neurons (Figure S10A-F), we next examined if skin derived 20-HETE is a major contributor of TRPV1-mediated itch and pain in CD disorder.

**Figure 8:**
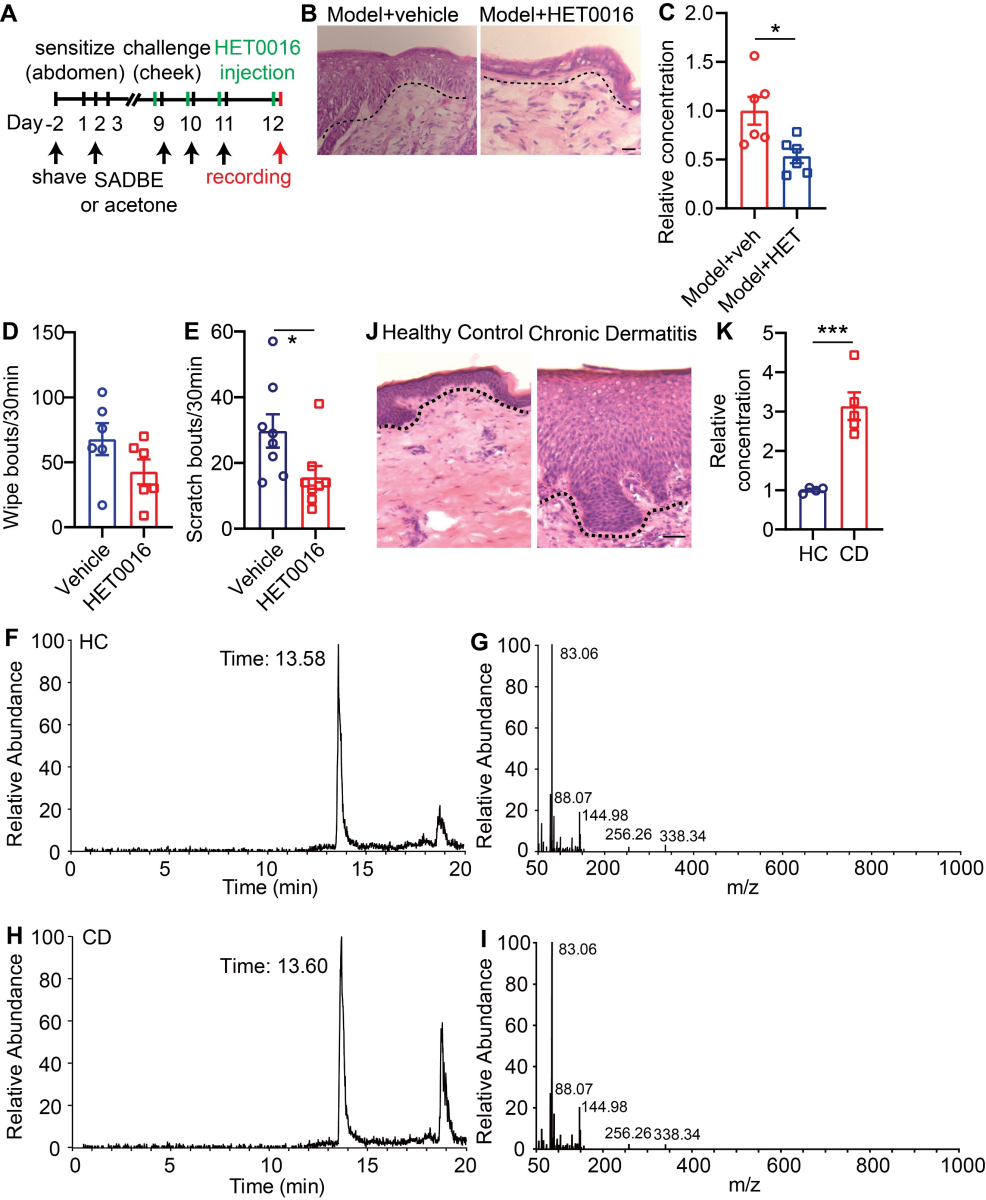
** 20-HETE mediated TRPV1 activation drives CD-associated itch. (A)** Schematic of the SADBE induced CD model and HET0016 administration. **(B)** Representative H&E staining of lesional skin from CD model mice treated with vehicle or HET0016. **(C)** Normalized 20-HETE abundance in lesional skin from CD model mice treated with vehicle or HET0016. **(D)** Spontaneous wiping in CD model mice treated with vehicle or HET0016. **(E)** Spontaneous scratching in CD model mice treated with vehicle or HET0016. **(F-I)** UHPLC ion chromatograms and MS spectra of 20-HETE in healthy control and lesional CD human skin. **(J)** Representative H&E staining of healthy control and lesional CD human skin. **(K)** Normalized 20-HETE abundance in healthy control and lesional CD human skin. *, *P* < 0.05; ***, *P* < 0.001. The scale bar represents 50 µm. All data are presented as mean ± SEM.

To accomplish this, wildtype CD model mice were given either vehicle or HET0016 (a selective 20-HETE synthase inhibitor 26) before each SADBE challenge (Figure 8A). Systemic HET0016 treatment blocked epidermal thickening at the SADBE challenged area (Figure 8B). ELISA assay confirmed that 20-HETE abundance was significantly reduced in the lesional skin of HET0016 treated mice (Figure 8C). Moreover, HET0016 treated mice showed mildly attenuated wiping behavior (68 ± 12.3 vehicle vs. 43 ± 9.6 HET0016) (Figure 8D), but significantly reduced scratching (30 ± 5.1 vehicle vs. 16 ± 3.5 HET0016) (Figure 8E). Finally, to explore if our findings in mice are relevant to human CD pathology, we tested 20-HETE availability in biopsies of healthy control (HC) and lesional CD skin by LC-MS (Figure 8F-I). Consistent with mouse CD model, lesional human CD skin showed significantly elevated 20-HETE abundance and prominent acanthosis, compared to HCs (Figure 8J-K).

## Discussion

Pain and itch are two fundamental sensory modalities initiated and mediated by primary sensory neurons. Recent progress has identified the discrete primary sensory neuron populations in the DRG and TG that detect, encode, and transmit pain and itch 6, 27. Mrgprs and the neurons that express them have been implicated as critical itch sensors, and as drivers of pathological itch that's commonly associated with chronic, inflammatory skin conditions. Primary sensory neurons that express MrgprA3 were recently reported to mediate both itch and pain - metabotropic activation of MrgprA3^+^ neurons evoked itch in mice, while ionotropic activation these neurons using optogenetics or ATP induced aversive responses distinct from scratching 28. While broad activation of TRPV1, an ion channel, by capsaicin results in burning pain, selective activation of MrgprA3^+^ neurons using capsaicin, in the absence of nociceptive neuron activation, evokes itch 13. The contribution of MrgprA3^+^ neurons and TRPV1 channels to CD-associated chronic itch and pain, however, is still unclear.

SADBE has been widely used to induce non-allergic and allergic CD in mice, SADBE model faithfully recapitulates skin inflammation and the chronic itch and pain symptoms of human CD, and was selected for studying CD-associated chronic itch and pain 14. Mrgprs were previously reported to be required for CD-associated itch 29. Our findings in current study further confirms that MrgprA3^+^ neurons become hypersensitive in CD condition, and drives CD-associated itch and itch hypersensitivity (Figures 2E). It should be noted that manipulation of MrgprA3^+^ neurons only affected itch related behavior in our CD model, as neither pharmacological activation of this population using CQ or silencing using hM4Di DREADD produced any detectible difference in pain related behaviors in CD model mice. These findings provide a potential mechanism for itch hypersensitivity commonly associated with human CD 30.

Remarkably, we found that capsaicin and TRPV1 actions become itch-biased in CD, leading to itch hypersensitivity. We found that capsaicin induced wiping behavior in both control and CD model mice, but wiping was not increased in CD model mice - suggesting that TRPV1 mediated pain is not changed in CD (Figure 1E). Conversely, capsaicin induced significant scratching in CD model mice, but not control mice, indicating that TRPV1 gains itch initiating functions in CD (Figure 1G).

Further studies using *Mrgprs^-/-^* mice suggested that, while spontaneous CD-associated itch is diminished by Mrgprs deficiency, capsaicin induced itch in CD model mice is not Mrgprs dependent (Figure 2B). This finding prompted us to instead examine the activity of MrgprA3^+^ neuron. Indeed, capsaicin induced scratching in CD model mice was abolished by DREADD silencing of MrgprA3^+^ neurons (Figure 2E). Our findings demonstrate that while Mrgprs are not required for CD itch hypersensitivity, MrgprA3^+^ neurons are. Additional experiments further showed that ERK phosphorylation was significantly increased in TGs, especially in MrgprA3^+^ neurons, of CD model mice. Previous studies have revealed that increased pERK, an indication of activated MAPK signaling, is related to increased neuronal excitability or stimuli sensitivity, and has been shown to result in the development of spontaneous chronic itch 23. To test if increased MrgprA3^+^ neuron excitability contributes to CD-associated itch hypersensitivity, we generated *MrgprA3;Braf* mice. In this line, constitutively active Braf is specifically expressed in MrgprA3^+^ neurons, resulting in dramatically elevated ERK phosphorylation in these neurons, increased MrgprA3^+^ neuron fiber density in the skin, and increased MrgprA3^+^ neuron excitability in response to current, CQ, and capsaicin stimulations (Figure 5F, H). *MrgprA3;Braf* mice were also scratched significantly more in response to CQ in the CD model. Importantly, capsaicin also induced scratching in *MrgprA3;Braf* mice, which was blocked by DREADD silencing (Figure 6D). These findings are consistent with the observations from the CD model mice and the notion that MrgprA3^+^ neuron hypersensitivity drives CD-associated alloknesis.

CD is always accompanied by skin barrier dysfunction and lipid metabolism disorders. The arachidonic acid metabolites 12-hydroperoxyeicosatetraenoyl acid (12-HpETE) 31 and 20-HETE 32 had emerged as potential endogenous activators of TRPV1. Our UHLPC and MS confirmed significantly increased 20-HETE abundance in lesional skin of CD model mice and human CD patients (Figure 7G, Figure 8K). Moreover, treatment with HET0016, a potent and selective 20-HETE synthase inhibitor, significantly attenuated 20-HETE availability in CD-like skin, as well as skin inflammation and itch (Figure 8B, C, E).

**Figure 9:**
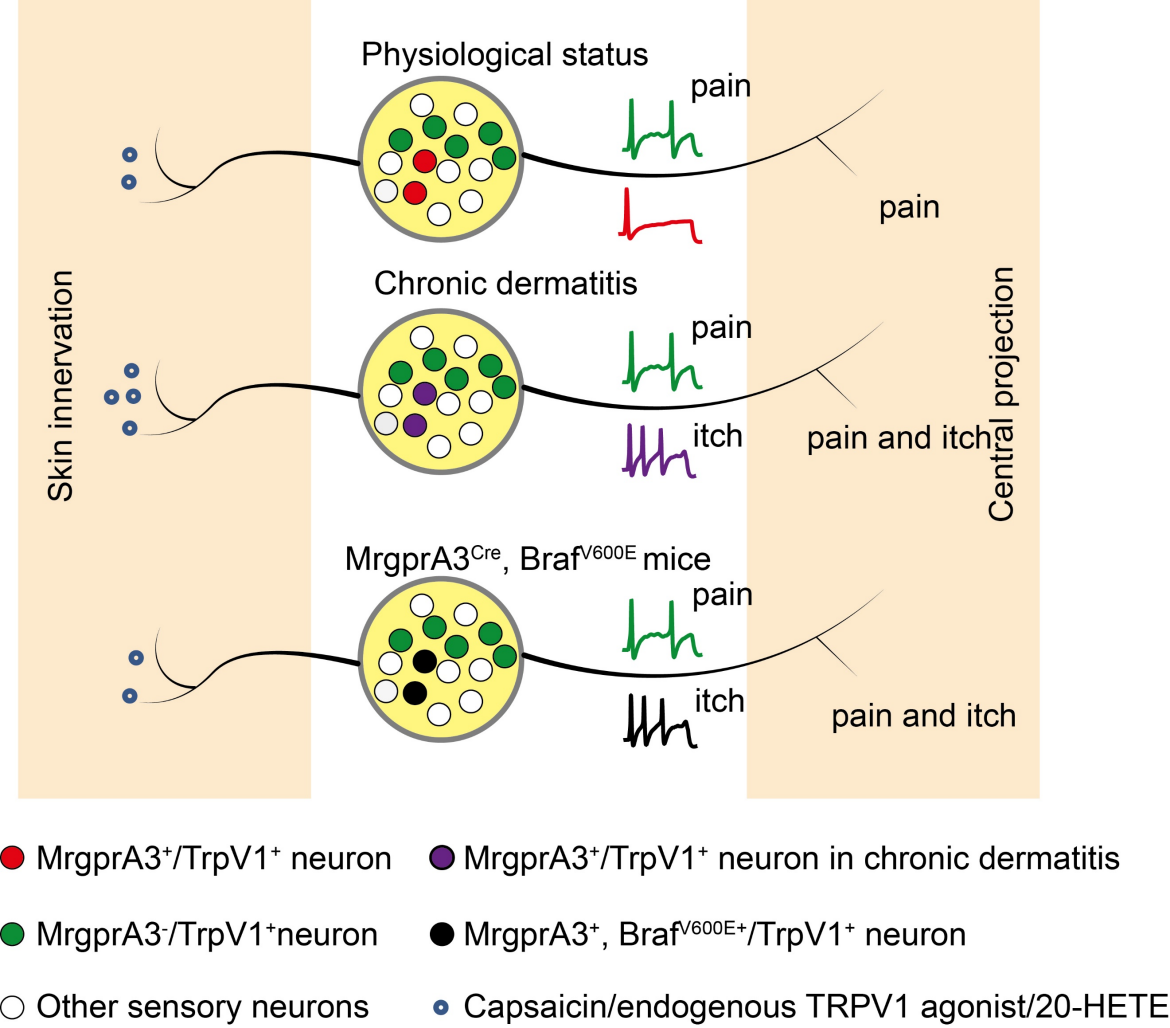
** TRPV1 mediated allokinesis in chronic dermatitis requires MrgprA3^+^ neuron hypersensitivity.** Chronic dermatitis enhanced the excitability of MrgprA3^+^ neurons (purple traces) and increased the abundance of 20-HETE, an endogenous TRPV1 ligand, resulting in allokinesis. Constitutively active Raf, leading to elevated ERK phosphorylation in MrgprA3^+^ neurons (black traces) mimics CD condition itch hypersensitivity.

Together, our study demonstrated that 1. pERK signaling is elevated in itch sensing MrgprA3^+^ neurons in CD condition, leading to hyperactivity of these neurons. And 2. the increased abundance of TRPV1 agonists in lesional CD skin leads to the over-activity of TRPV1 channels in hyperactive MrgprA3^+^ neurons, resulting in TRPV1 mediated allokinesis (Figure. 9). Moreover, we demonstrated that silencing MrgprA3^+^ neurons were an effective strategy for itching relief in mouse CD model. Our study elucidated a critical mechanism of CD-associated chronic itch and pain. These finding advanced our understanding CD pathology as well as sensory biology, and identified novel targets for chronic itch management in clinics.

## Supplementary Material

Supplementary figures.

## Abbreviations

TRPV1: Transient Receptor Potential Vanilloid 1
MrgprA3: Mas-related G protein-coupled receptor A3
TG: trigeminal ganglia
DRG: dorsal root ganglia
CD: chronic dermatitis
SADBE: squaric acid dibutylester
CHS: contact hypersensitivity
Aps: action potentials
DREADDs: designer receptors exclusively activated by designer drugs
CNO: clozapine N-oxide
