# Supplementary material for: 20-HETE mediated TRPV1 activation drives allokinesis via MrgprA3+ neurons in chronic dermatitis
Source: Theranostics. 2024 Feb 4;14(4):1615–30. doi: 10.7150/thno.85214 (PMC10879873; doi:10.7150/thno.85214)
Supplement: Supplementary file 1 — Supplementary figures. [file thnov14p1615s1.pdf]

Supplemental figures

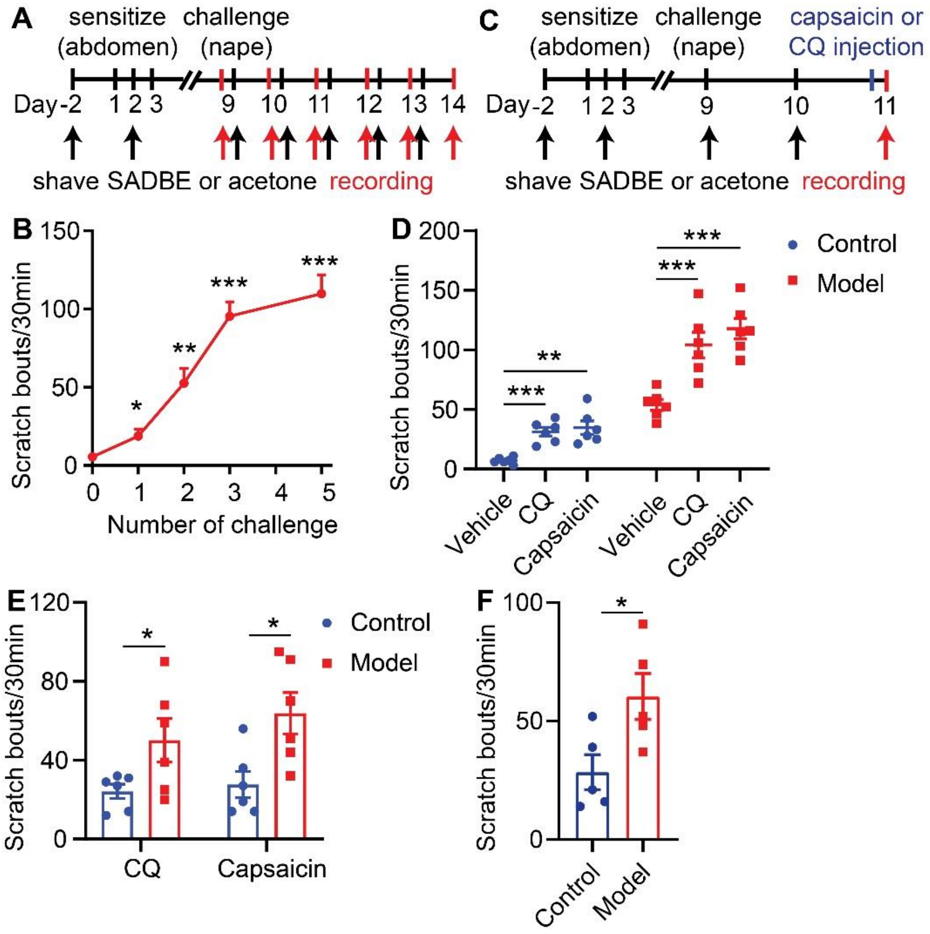

Figure S1. SADBE mice showed increased sensitivity toward CQ or capsaicin. (A) Schematic of the SADBE induced nape CD model. **(B)** Spontaneous scratching behavior in SADBE treated CD mice. (*, compared with day 0; n = 6). **(C)** Schematic of the SADBE induced nape CD model and time points of drug injections. CQ or capsaicin were nape inject 24 hours after two challenges. **(D)** CQ or capsaicin induced scratching behavior in control and CD mice (n = 6). **(E)** Change in scratching response after CQ or capsaicin injection in control and CD model mice. (n = 6). **(F)** Change in scratching response after capsaicin injection in control and CD model female mice. (n = 5). *, *P* < 0.05; **, *P* < 0.01; ***, *P* < 0.001. All data are presented as mean ± SEM.

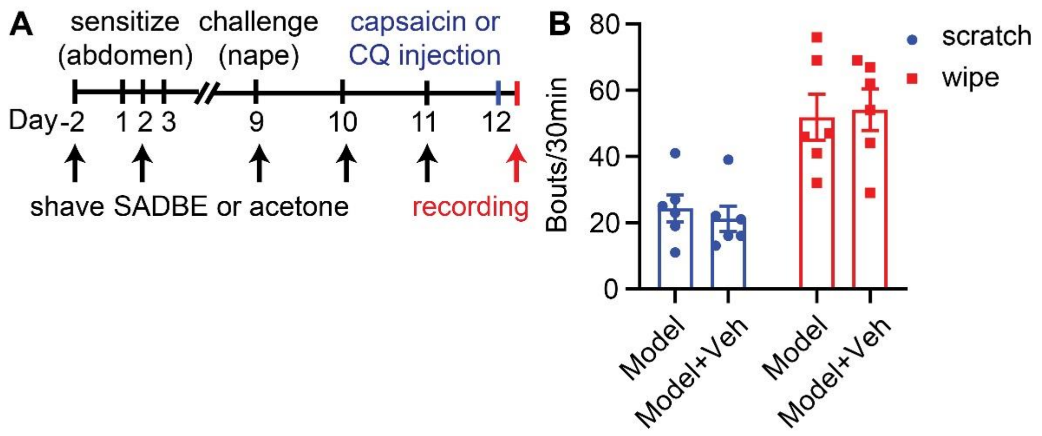

Figure S2. Schematic of the cheek model experiment. (A) Schematic of the SADBE induced cheek CD model and time points of drug injections. CQ or capsaicin were cheek inject 24 hours after three challenges. **(B)** Vehicle injection did not affect scratching or wiping behavior in CD model mice.

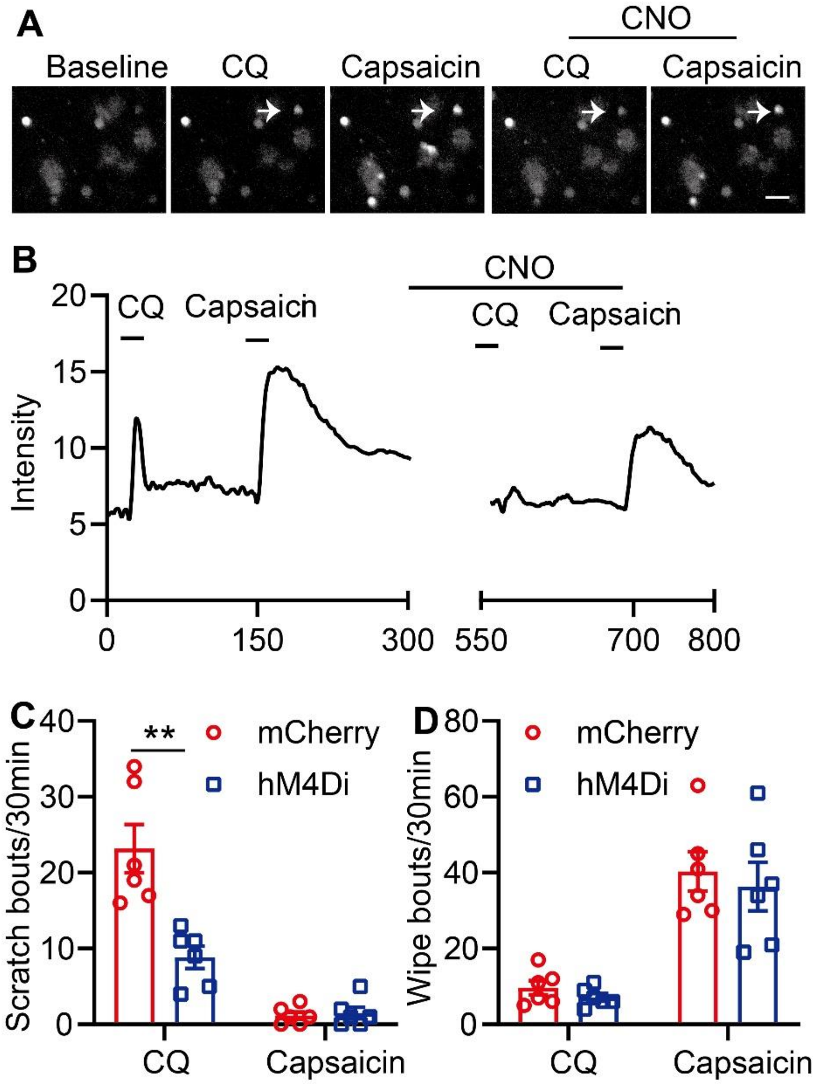

Figure S3. CQ induced calcium response in cultured TG neurons and scratching behavior in mice were inhibited in the hM4Di group after CNO pretreatment. (A-B) CQ induced calcium response in cultured TG neurons was decreased in the hM4Di group after CNO pretreatment. **(C)** CQ or capsaicin induced scratching behavior in control and MrgprA3-Gi DREADD expressing mice after CNO pretreatment. (n = 6). **(D)** CQ or capsaicin induced wiping behavior in control and MrgprA3-Gi DREADD expressing mice after CNO pretreatment. The scale bar represents 50 µm. **, $P < 0.01$. All data are presented as mean ± SEM.

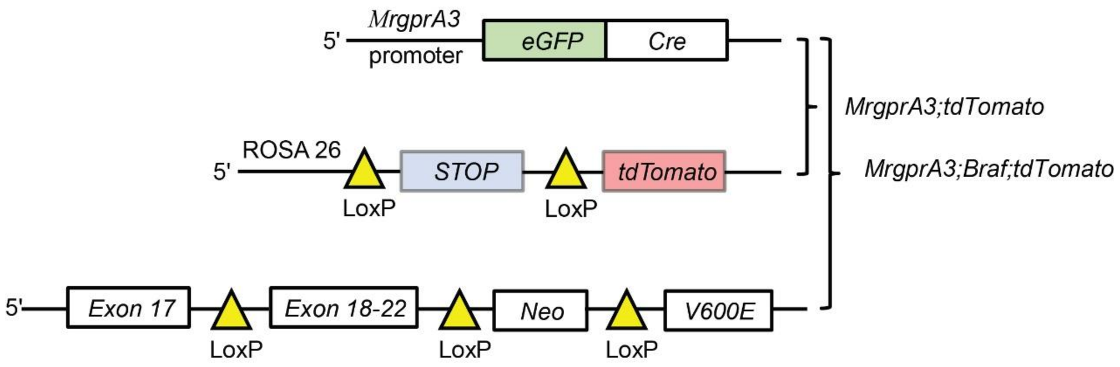

Figure S4. Schematic of *MrgprA3;Braf* and *MrgprA3;Braf;tdTomato* mice.

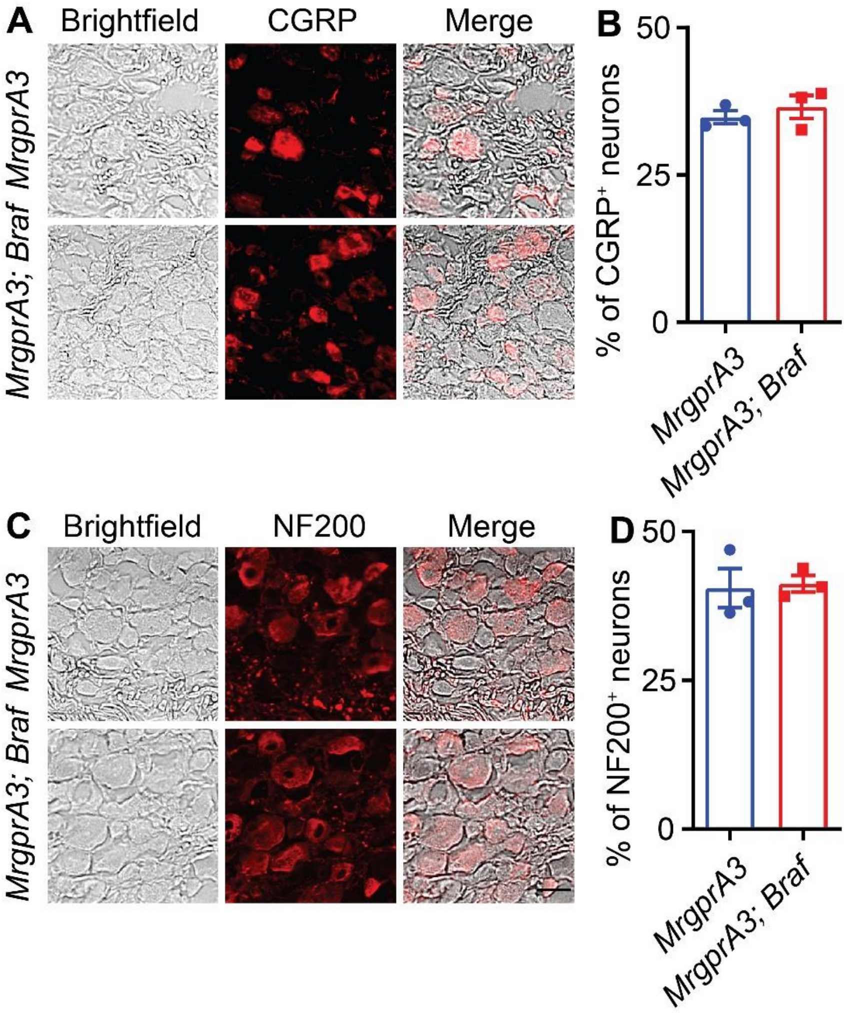

Figure S5. The proportion of CGRP⁺ and NF200⁺ TG neurons was not changed in *MrgprA3;Braf* mice. (A-B) Immunostaining and quantification of CGRP⁺ neurons in TGs from control *MrgprA3-Cre* and *MrgprA3;Braf* mice. **(C-D)** Immunostaining and quantification of NF200⁺ neurons in TGs from *MrgprA3-Cre* and *MrgprA3;Braf* mice. The scale bar represents 25 µm. All data are presented as mean ± SEM.

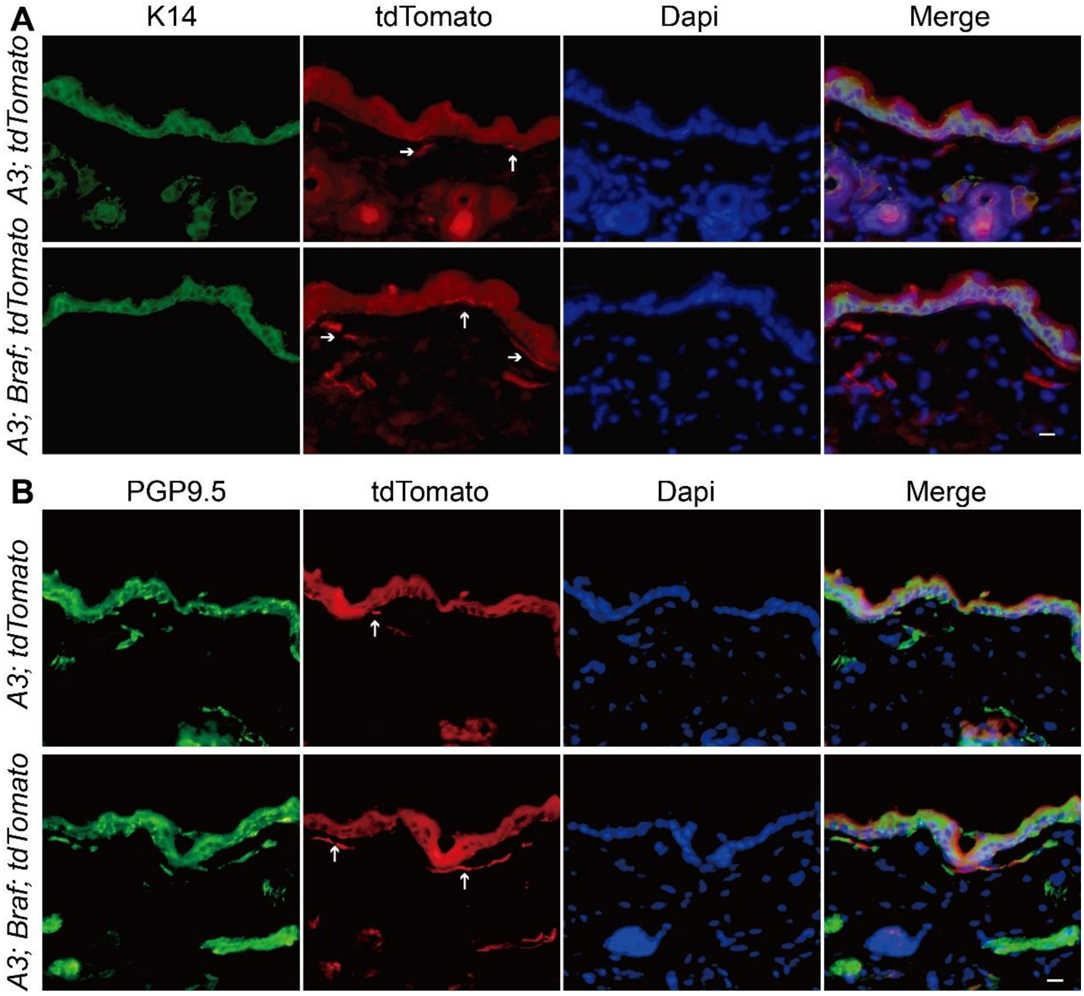

Figure S6. MrgprA3^{+} skin fiber, K14 and PGP9.5 staining. (A) Immunostaining of the keratinocyte marker K14 and tdTomato in skin of *MrgprA3;tdTomato* and *MrgprA3;Braf;tdTomato* mice. **(B)** Immunostaining of the pan neuronal marker PGP9.5 and tdTomato in skin of *MrgprA3;tdTomato* and *MrgprA3;Braf;tdTomato* mice. The scale bar represents 25 µm.

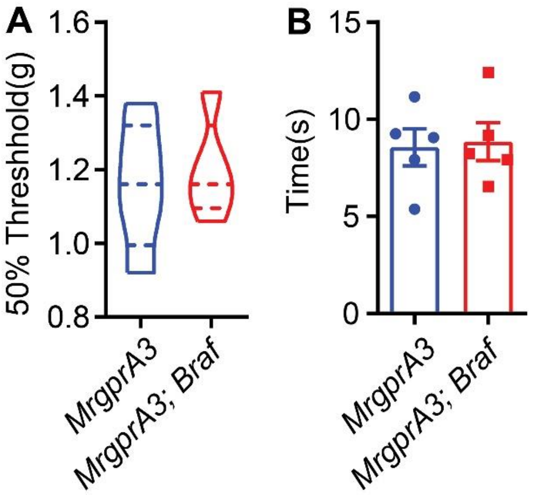

Figure S7. Enhanced ERK signaling in MrgprA3^{+} neurons does not affect acute pain behavior. (A) Mechanical pain response of control *MrgprA3-Cre* and *MrgprA3;Braf* mice. **(B)** Thermal pain response of control *MrgprA3-Cre* and *MrgprA3;Braf* mice. All data are presented as mean ± SEM.

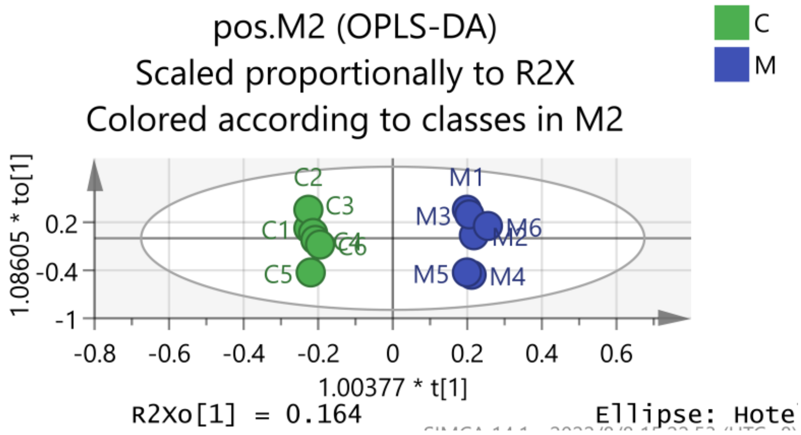

Figure S8. OPLS-DA score plots of the control mice (C) and CD model mice (M).

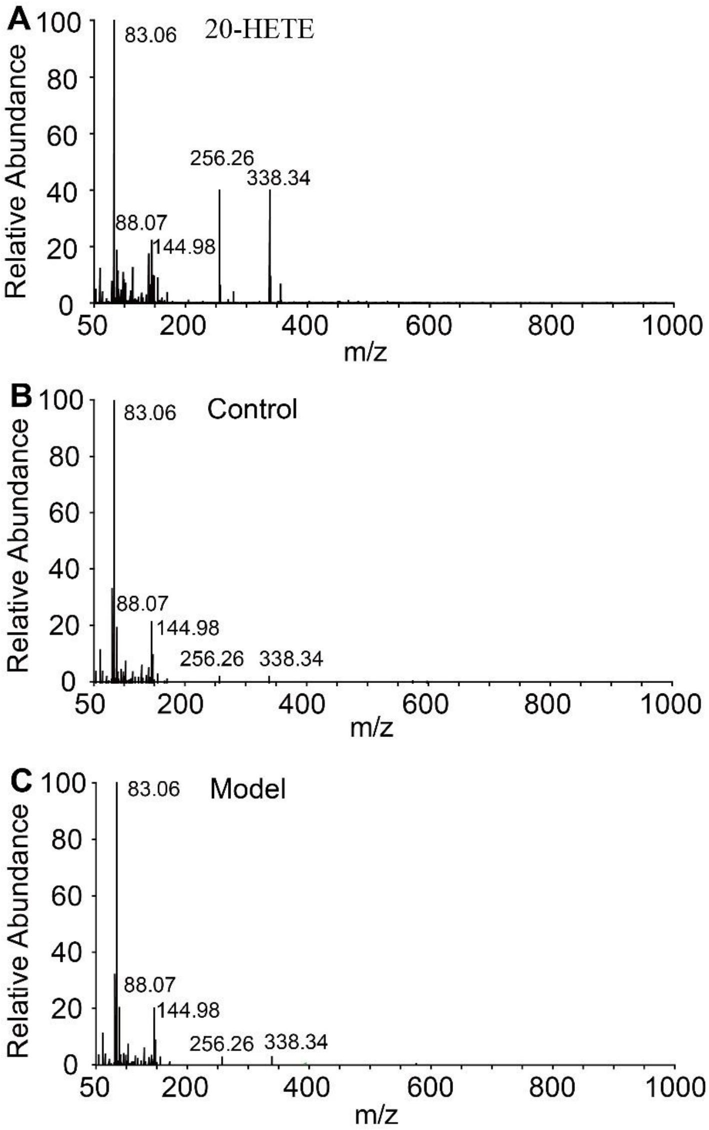

Figure S9. The MS spectrum of 20-HETE in control and lesional skin of CD model mouse. (A) The MS spectrum of reference 20-HETEcompound (Cayman). **(B-C)** The MS spectrum of 20-HETE in control mouse skin lysate and SADBE treated lesional skin lysates.

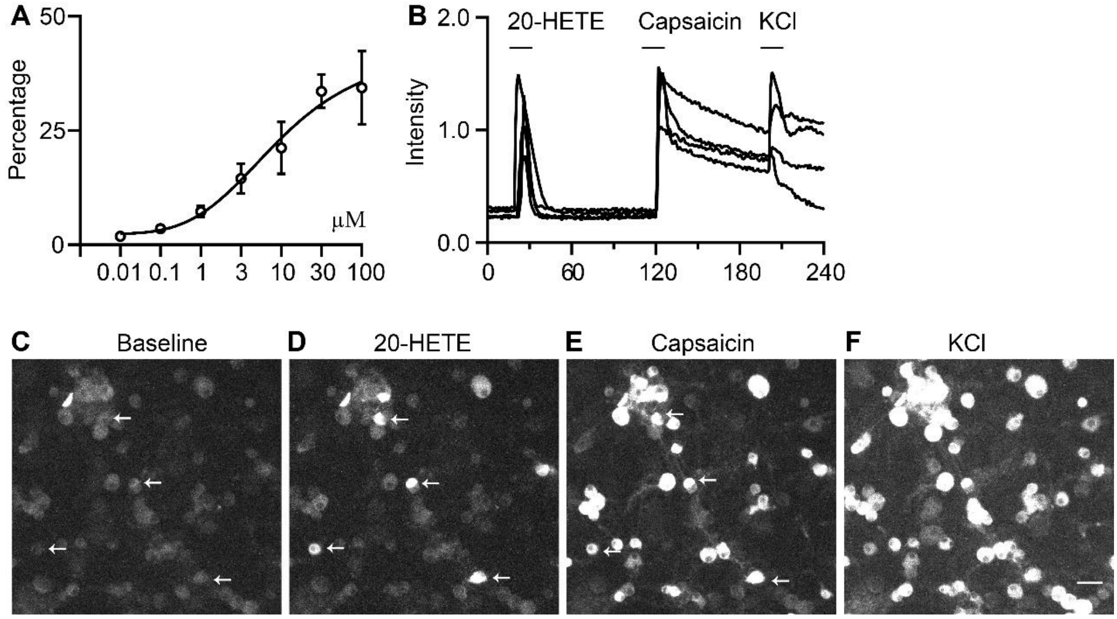

Figure S10. 20-HETE induces calcium influx in TRPV1⁺ TG neurons. (A) Dose response curve of TG neurons to 20-HETE. **(B)** Representative calcium response traces of TG neurons in response to 20-HETE, capsaicin, and KCl. **(C-F)** Representative images of calcium responses of TG neurons to 20-HETE (1 µM), capsaicin (1 µM) and KCl (1 mM). The scale bar represents 50 µm. The scale bar represents 25 µm.
